# Antioxidant Potential of Jostaberry Phytochemicals Encapsulated in Biopolymer Matrices During Storage

**DOI:** 10.3390/foods14173092

**Published:** 2025-09-03

**Authors:** Angela Gurev, Viorica Bulgaru, Veronica Dragancea, Olga Smerea, Alexei Baerle, Georgiana Gabriela Codină, Aliona Ghendov-Mosanu

**Affiliations:** 1 Faculty of Food Technology, Technical University of Moldova, 9/9 Studentilor St., MD-2045 Chisinau, Moldova; angela.gurev@chim.utm.md (A.G.); viorica.bulgaru@tpa.utm.md (V.B.); veronica.dragancea@chim.utm.md (V.D.); olga.smerea@doctorat.utm.md (O.S.); alexei.baerle@chim.utm.md (A.B.); 2 Faculty of Food Engineering, Stefan cel Mare University, 720229 Suceava, Romania

**Keywords:** josta, alginate, maltodextrin, nutriose, pectin, polyphenols, anthocyanins, antioxidant activity

## Abstract

The jostaberry (*Ribes × nidigrolaria*) hybrid is a rich source of phytochemicals with high antioxidant activity (AA). However, due to the thick skin and seeds, the whole fruits are rejected by some consumers, and their incorporation into food products may negatively affect the sensory properties. Furthermore, after drying, including freeze-drying, jostaberries become sticky and gummy, making them unsuitable for grinding into powder. In this context, the present study aims to improve the handling properties and evaluate the biological value, antioxidant potential, physicochemical characteristics, and color parameters of biopolymer microparticles enriched with biologically active compounds (BACs) from jostaberry during freeze-drying and subsequent storage in the dark under ambient conditions (22 ± 1 °C, relative humidity ≤ 75%). For this, jostaberry extract (JE) was encapsulated using combinations of biocompatible carriers: maltodextrin-nutriose (resistant dextrin)-pectin and maltodextrin-nutriose-sodium alginate. The encapsulated products were freeze-dried to obtain microparticles (MNPJ and MNAJ) with yields of 87.7% and 88.9%, respectively. It was found that the biopolymer matrix provided superior protection for the encapsulated BACs during freeze-drying compared to the fruit matrix. The AA determined in MNPJ and MNAJ microparticles by DPPH and ABTS assays decreased only 1.1 and 1.5 times, respectively, while in freeze-dried jostaberry, AA showed a decrease of 3.7 times (DPPH) and 2.3 times (ABTS), respectively. Tukey’s post hoc HSD analysis revealed multiple significant differences (*p* < 0.05) between storage intervals for all measured parameters. While DPPH and ABTS values progressively decreased, total polyphenols (TPC) and anthocyanins concentration (TAC) and their retention efficiency showed changes after specific storage intervals (3, 6 and 12 months). After 12 months of storage, TPC and TAC decreased by 8.2% and 12.2% in MNPJ and by 3.3% and 3.9% in MNAJ, respectively. Therefore, microparticles containing sodium alginate showed the lowest BAC loss during storage. The obtained results reveal that after 12 months of storage, the color and physicochemical properties of the microparticles remained largely stable.

## 1. Introduction

Nowadays, consumer preferences are increasingly shifting towards foods containing natural and sustainable ingredients. At the same time, stricter European Union legislation is stimulating the use of natural additives in the food industry, especially plant-derived phytochemicals such as colors, antioxidants, preservatives, emulsifiers, stabilizers, gelling agents, thickeners, sweeteners, and flavors [1]. Some of the richest sources of biologically active compounds (BACs) are berries, including the hybrid Josta varieties [2,3], which combine the characteristics and phytochemical properties of the species from which it comes: blackcurrant and gooseberry [4]. Jostaberries are rich in organic acids, polyphenols [5] including flavonoids (over 50 flavonols have been identified) [6], and minerals, fibers and other compounds. BACs in berries have health benefits for the consumer, reduce oxidative stress in the body, protect the cardiovascular system [7], have anticancer activity [8], immunomodulatory, anti-inflammatory [9], antimicrobial and antifungal [10], antidiabetic [11], neuroprotective [12], and other aspects. Berry powder and extracts are increasingly used as food supplements, natural additives and ingredients for functional foods [13].

Research has shown that unfortunately, a number of BACs from plant sources are not stable in food environments [14]. Flavonoids, including anthocyanins, vitamins and pigments degrade, losing their antioxidant activity (AA) under various unfavorable conditions, during food processing and storage, at high temperatures, in the presence of oxidative enzymes, pH changes, light, humidity, the presence of oxygen and during gastrointestinal digestion [15,16]. Sadowska et al. reported that freezing berries at −18 °C resulted in vitamin C and anthocyanin losses of 14% and 10%, and during freeze-drying and air-drying, anthocyanin losses, compared to fresh fruits, were 82% and 94%, respectively [17].

Several studies have demonstrated that BAC encapsulation in biopolymer matrices is an emerging process that enhances stability and protects sensitive components from adverse environmental conditions [18], while ensuring the controlled release of bioactive compounds [19]. According to the literature, numerous methods have been developed for the encapsulation of bioactive compounds in various carriers, especially biopolymers compatible with the human body. The most commonly used encapsulation matrices are dietary fibers, approved as food additives in the European Union [20], known for their prebiotic effects. These include starch, dextrins, pectin, cellulose, alginate, chitosan and others [21,22]. They can be applied individually or as combinations of different biopolymers. It was reported that the combination of wall materials resulted in increased stability of anthocyanins, protecting them even under simulated gastrointestinal conditions, which was possibly due to synergistic action [23,24].

The encapsulated materials were dried using different techniques, including air-drying, oven-drying [25], freeze-drying [26], supercritical CO_2_ drying [27] and spray-drying [28]. Among these, spray-drying and freeze-drying generally offer higher yields and encapsulation efficiency. In particular, several studies reported a significantly higher content of anthocyanins in freeze-dried samples compared to spray-dried samples [29,30], volatile compounds and heat-sensitive phytochemicals were also protected [31].

Recent studies [5] indicate that the phytochemical profile of jostaberry is influenced by processing methods (freezing, drying, freeze-drying) and extraction techniques. Drying, especially oven drying, alters the color, appearance, phytochemical content of berries, as well as AA. After drying, including freeze-drying, jostaberries become sticky and cannot be ground into powder. Freeze-drying also reduces the concentration of sensitive BACs; consequently, the AA measured in freeze-dried jostaberry extracts decreased by more than half compared to that of frozen fruit. Other studies have shown that the drying method significantly affects the physicochemical properties, appearance and BAC content of berries [32]. Pateiro et al. [33] reported that the air-drying of jostaberry at 103 °C for 4 h resulted in crust formation, development of undesirable aromas and browning reactions.

Encapsulation of jostaberry extracts in wall materials, such as polysaccharides [18], can enhance physicochemical stability and preserve the biological potential and antioxidant activity of phytochemicals, while improving handling and storage. In addition, the introduction of anionic polysaccharides into the wall material could better retain and protect BACs through electrostatic interactions, which in the case of sodium alginate could be more advanced.

At the same time, there is less data in the literature regarding the influence of freeze-drying and storage time on the biological value and AA of plant extracts encapsulated in combined polysaccharide matrices.

The aim of this study was to improve the handling properties and evaluate the biological value, antioxidant potential, physicochemical properties and color parameters of biopolymer microparticles containing jostaberry phytochemicals during freeze-drying and storage in the dark, under ambient conditions. To achieve this, jostaberry extract (JE) was microencapsulated using combinations of biocompatible carrier agents: maltodextrin-nutriose (resistant dextrin)-pectin (MNP) and maltodextrin-nutriose-sodium alginate (MNA). The microencapsulated products were freeze-dried to obtain stable, water-soluble microparticles (MNPJ and MNAJ), and their biological potential was evaluated during manufacturing and after 3, 6 and 12 months of storage.

## 2. Materials and Methods

### 2.1. Chemicals

The 2,2-azinobis (3-ethylbenzothiazoline-6-sulfonic acid), diammonium salt (ABTS) (purity ≥ 98%), 2,2-diphenyl-1-picrylhydrazyl-hydrate (DPPH) (≥95%), 6-hydroxy-2,5,7,8-tetramethylchromane-2-carboxylic acid (Trolox) (≥97%) were provided by Alpha Aesar (Haverhill, MA, USA). Folin–Ciocalteu phenol reagent (2.1 N) was purchased by Chem-Lab NV (Zedelgem, Belgium). Gallic acid (GA) (≥97%), aluminum chloride hexahydrate (≥98%), rutin (≥94%), quercetin,, cyanidin-3-*O*-glucoside ascorbic acid, citric acid, fumaric acid (≥95%); chlorogenic acid, caffeic acid and malic acid (≥97%), were purchased by Sigma-Aldrich (St. Louis, MO, USA). Reagents of chromatographic and analytical purity were utilized throughout the experiments. Spectrophotometric determinations were conducted on a UV-1900 spectrophotometer (Shimadzu, Tokyo, Japan). High-Performance Liquid Chromatography with Photo Diode Array (HPLC-PDA) detection was performed on Shimadzu LC 2030C 3D Plus (Shimadzu, Tokyo, Japan) using reversed-phase C18-column Phoenomenex (150 mm × 4.6 mm × 4 μm) and 2-phase gradient elution.

### 2.2. Vegetable Material and Carrier Encapsulation Agents

Jostaberries (*Ribes* × *nidigrolaria*) were collected from a local supplier at the end of July 2023. After sorting, washing and drying, the berries were frozen. at −29 ± 1 °C for 10 h, then packed and stored at −19 ± 1 °C. After a storage period exceeding three months under freezing conditions, the dry matter content of frozen jostaberries (FJ) was determined.

For carrier agents, plant-derived biopolymers that are safe and biocompatible with the human body were selected (Table 1).

**Table 1:** Characteristics of encapsulation carrier agents.

Biopo2lymers	Characteristics
Maltodextrin	Maltodextrin with a food-grade DE of 18, certified under ISO 9001:2015 [34] and FSSC 22000, was supplied by Interstarch, Ukraine. It is a water-soluble polysaccharide produced by the partial enzymatic hydrolysis of starch. Recognized as a natural food additive, maltodextrin’s higher DE value corresponds to faster assimilation by the human body. As a carrier agent, it helps reduce the stickiness of plant extract powders during drying [35].
Nutriose	Water-soluble dextrin, derived from highly branched corn starch, was marketed under the trade name Nutriose^®^ by Gesundo, GM Gesundheits Manufactur GmbH, Germany. It is a highly branched polysaccharide composed of glucose units linked by 1–6, 1–2, and 1–3 glycosidic bonds. Due to this chemical structure, only approximately 10–15% of the resistant dextrin units are broken down in the stomach and small intestine, while the remainder is progressively fermented in the colon, providing a prebiotic effect [36]. Supplementation with Nutriose for 12 weeks has been shown to promote satiety, improve intestinal transit, and reduce body weight [37].
Sodium alginate	Sodium alginate (100% food grade) was supplied by Shandong Jiejing Group Corporation, China. It is a natural anionic polysaccharide biopolymer derived from brown marine algae. Sodium alginate is widely used for its ability to eliminate toxins and heavy metals from the body [38], and as a carrier for drugs and bioactive substances due to its excellent biocompatibility [39].
Pectin	Commercial pectin GRINDSTED^®^ Pectin MRS 351 from DuPont Danisco (DE > 60%) is a heteropolysaccharide and a natural food additive (E440), serving as a source of soluble dietary fiber. It is obtained from fruits or vegetables. Pectin can protect encapsulated materials during digestion due to its resistance to proteases and amylase, which are active in the upper gastrointestinal tract. Partially degraded by colonic microflora, pectin exhibits a prebiotic effect, helps eliminate toxins, reduces cholesterol, and has anti-inflammatory properties [40]. Additionally, it serves as a carrier for medications and BACs [41].

### 2.3. Obtaining JE for Encapsulation

Extracts for encapsulation were prepared in triplicate from FJ as follows: 100 g of FJ were blended with an aqueous solution of 70% ethanol at a sample-to-solvent ratio of 1:3 (*m*/*v*). The mixture was macerated at 4 °C in the dark for 16 h, followed by ultrasound-assisted extraction (USAE) using an ISOLAB device (Laborgeräte GmbH, Eschau, Germany) at a frequency of 37 kHz and a temperature of 35 ± 1 °C for 20 min. The extract was then filtered, and the residue was extracted a second time with another portion of hydroalcoholic solution (1:3, *m*/*v*) under the same conditions. Following centrifugation of the combined extracts at 9500 rpm for 20 min, the supernatants were recovered. Ethanol was removed by vacuum evaporation at 40 ± 1 °C and 175 mbar pressure. Total soluble solids (TSS) in the JE were measured at 20 °C using a digital refractometer (PT-32, ATAGO, Tokyo, Japan) following AOAC (2005) methods and expressed in degrees Brix (°Bx).

The determination of total solids (TS) was carried out as described in AOAC (2005), by evaporating 10 g of JE at 105 ± 1 °C in an oven. The residue was weighed, and TS values were calculated, and expressed in %. The total polyphenol content (TPC), total anthocyanin content (TAC), and AA of JE were determined by the methods described below.

### 2.4. The Microencapsulation and Freeze-Drying Process

To prepare the MNP hydrogel, maltodextrin (30%) was dissolved in 60 mL of distilled water, followed by the addition of nutriose (8%) and then pectin (2%), with continuous stirring. Similarly, the MNA hydrogel was prepared by replacing pectin with sodium alginate (2%). Each hydrogel (100 mL) was allowed to hydrate at 4 °C for 24 h. Each type of hydrogel was prepared in three replicates.

The hydrogels were then homogenized by stirring at 140 rpm and 30 °C for 30 min in a water bath. While stirring continuously, 100 mL of JE was added to each hydrogel, resulting in ratio of solids in JE versus total carrier solids of approximately 1:2 (*w*/*w*). The loaded hydrogels (MNPJ and MNAJ) were stirred continuously at 30 °C for 20 min.

The hydrogels (MNPJ, MNAJ) and FJ were freeze-dried using a Christ Gamma 2/16 LSC plus freeze-dryer. The freeze-drying process was conducted in two stages: the first stage at 13 Pa pressure, −29 ± 1 °C for 10 h; the second stage at 13 Pa pressure, 19 ± 1 °C for 10 h.

After freeze-drying, the products were weighed, slightly crushed into microparticles, and packaged in dark glass container, into which a silica gel packet was inserted. A sealing foil was applied to the mouth of the container, and it was hermetically closed with a screw cap with internal gaskets. The samples of freeze dried jostaberry (FDJ) were sticky and could not be ground. The microparticles were stored in the dark, in ambient conditions (22 ± 1 °C and relative humidity max. 75%). The storage conditions were chosen based on the storage conditions of food supplements or pharmaceuticals on store shelves.

### 2.5. Physicochemical Analysis of Jostaberries and Microparticles

Moisture content (MC), dry matter (DW), fat content (FC) and ash content (AC) were measured according to the AOAC (2012) method.

### 2.6. Evaluation of Color Parameters

The color of the samples was evaluated using a Chroma Meter CR-400 (Konica Minolta, Japan). Results were expressed according to the CIELab system, providing values for lightness (L*), the a* coordinate (red to green), and the b* coordinate (yellow to blue). Based on these primary parameters, chromaticity (C*) and hue angle (h*) were derived using established formulas [42].

By changing the pH in the aqueous solutions of microparticles (in a microparticle:distilled water ratio of 1:10, (*m*/*v*)) the red color range was determined.

### 2.7. Microparticles Production Efficiency (MPE)

MPE was expressed in percentages calculated as the ratio of the mass of microparticles obtained to the total mass of solids in the feed (in terms of dry weight (DW)).

(1) $$MPE=\frac{mass\;of\;microparticles\;(DW)}{mass\;of\;all\;solids\;\left(DW\right)\;in\;the\;feed}\times 100\%,$$

### 2.8. Bulk Density (BD), TSS, Hygroscopicity, pH, Titrable Acidity (TA)

BD was assessed as per the method available in the bibliography [43].

TSS content was determined by dissolving 5 g of MNPJ and MNAJ microparticles in 50 mL of distilled water. The mixture was centrifuged at 8000× *g* for 25 min to obtain the supernatant. TSS was measured using a digital refractometer and expressed in °Bx.

The supernatant was filtered, and its pH was measured. TA was determined through titration of the supernatant until pH 8.2, with values reported as % citric acid.

To determine hygroscopicity, the method of Sarabandi et al. [44] with some modifications was applied. Two grams of sample were placed in a desiccator containing a saturated sodium chloride solution (75% relative humidity). Hygroscopicity was determined after 24 h by weighing the samples and expressing the results as g of absorbed water per 100 g of dry solids.

### 2.9. Solubility and Oil Holding Capacity (OHC)

The solubility of the microparticles was determined following the method of Cani and Chauca et al. [45].

OHC was determined as follows: 25 mL of vegetable oil was added to 5 g of microparticles. The mixture was vortexed for 30 s and then centrifuged at 1300× *g* for 25 min at 25 °C. The residue was weighed, and the absorbed oil mass was determined. OHC was expressed as a percentage and calculated according to Equation (2).

(2) $$OHC=\frac{soaked\;sample\;mass-initial\;sample\;mass}{initial\;sample\;mass}\cdot 100\%$$

### 2.10. Phytochemical Composition and AA

#### 2.10.1. BAC Extraction for Spectrophotometric Analysis

BACs from FJ and FDJ were extracted using a 70% hydroethanolic solution in accordance with source [5]. Extraction was performed using ultrasound-assisted extraction (USAE) at 37 kHz frequency and 35 °C for 20 min.

For spectrophotometric analysis, BAC extraction from MNPJ and MNAJ microparticles was carried out in the following manner: 0.5 g of sample was hydrated with 5 mL of distilled water by vortexing for 1 h. Then, 20 mL of 87.5% aqueous ethanol was added to reach a final ethanol concentration of 70%, with a sample-to-solvent ratio of 1:50 (*m*/*v*). The tubes were vortexed for 60 min at 25 °C and then centrifuged at 10,000 rpm for 10 min. The supernatants were collected for analysis.

Extracts from loaded MNPJ and MNAJ hydrogels (encapsulated materials before freeze-drying) were prepared by adding 23.3 mL of 70% hydroethanolic solution to 1.7 g of hydrogel, maintaining a solid-to-liquid ratio of 1:50 (*m*/*v*). After vortexing the mixture for 60 min at 25 °C, it was centrifuged at 10.000 rpm for 10 min. The supernatants were then analyzed.

#### 2.10.2. Determination of TPC and Total Flavonoid Content (TFC)

The TPC and TFC in the hydroalcoholic extracts of FJ, FDJ, and MNPJ and MNAJ hydrogels and microparticles were determined spectrophotometrically using methods described in the literature [46], with minor modifications [47].

TPC was assessed according to the Folin–Ciocalteu method [48], applying a gallic acid standard curve (0–500 mg/L, R^2^ = 0.9977) and expressing the outcomes as mg gallic acid equivalents per g of dry weight (mg GAE/g DW). TFC was determined using AlCl_3_·6H_2_O, according to calibration curves with quercetin (0–160 mg/L, R^2^ = 0.9972) and rutin (0–160 mg/L, R^2^ = 0.9991). The results were expressed as mg rutin equivalents (RuE)/g DW and mg quercetin equivalents (QE)/g DW, respectively.

#### 2.10.3. Determination of TAC

The pH-differential method described by Lee et al. [49] was applied for the determination of TAC in hydroalcoholic extracts. For this purpose, the extracts were mixed with buffer solutions at pH 1.0 (KCl) and pH 4.5 (CH_3_COONa), and absorbance was measured at 520 nm and 700 nm. TAC was quantified and expressed in terms of mg cyanidin-3-*O*-glucoside equivalents (Cy3GE)/g DW.

#### 2.10.4. Retention Efficiency (RE) of Polyphenols and Antocyanins

RE of jostaberry phytochemicals in the biopolymer matrix of freeze-dried microparticles was calculated as a percentage (%) of the ratio of TPC or TAC determined in microparticles (mg GAE/g DW) to TPC or TAC determined in the encapsulated materials before freeze-drying (mg Cy3GE/g DW).

#### 2.10.5. Assessment of DPPH Free Radical Scavenging Activity

The AA was determined using the Trolox equivalent antioxidant capacity (TEAC) assay based on DPPH• radical scavenging, as described in the literature [50]. Values were reported as mg trolox equivalents (mg TE/g DW) using a calibration curve of 0–300 µM (R^2^ = 0.9992) with trolox. Samples with higher concentrations were appropriately diluted before measurement.

#### 2.10.6. Assessment of ABTS Free Cation-Radical Scavenging Activity

To evaluate the ability of extracts from FJ, FDJ, and MNPJ and MNAJ hydrogels and microparticles to scavenge the ABTS•^+^ cation-radical in the TEAC assay, the method of Arnao et al. [51] was followed. Values were reported as mg TE/g DW, based on a calibration curve with trolox (0–500 µmol/L, R^2^ = 0.9996).

#### 2.10.7. HPLC-PDA Detection

HPLC-PDA analysis was realizedin accordance with the method described by Bulgaru et al. [5].

Prior to being analyzed, FJ and FDJ extracts (70% hydroethanol, 1:100 *m*/*v*) were passed through 0.22 μm polyethersulfone filters.

#### 2.10.8. Quantitative Analysis of Organic Acids

Capillary electrophoresis, as described in [52], was used to quantify the total organic acids in hydroethanolic extracts of FJ and FDJ (1:100, *m*/*v*), with values expressed in mg/g.

### 2.11. Microstructure Analysis of Microparticles

The microstructure and surface morphology of the microparticles were analyzed using a TESCAN Vega TS 5130 MM scanning electron microscope (SEM), following the method described in the literature [53]. The limiting diameter of the Au dots was 20 ± 2 nm. The samples were then examined at 80× and 1000× magnifications, at 10.0 kV accelerating voltage, and the resulting images were processed for analysis.

### 2.12. Statistical Analysis

Data are expressed as mean ± standard error of the mean from three independent measurements. Statistical analyses were performed using Microsoft Excel 2007 (Microsoft, Redmond, WA, USA) and Statgraphics Centurion XVI 16.1.17 (Statgraphics Technologies, Inc., The Plains, VA, USA). Differences between groups were evaluated by one-way ANOVA followed by Tukey’s post hoc test, with significance set at *p* < 0.05. The principal component analysis (PCA) and Person correlation were performed using Scikit-learn Python library to determine the relationship between microparticle samples and the quality attributes [54].

## 3. Results and Discussion

### 3.1. Jostaberry and Jostaberry Extracts Properties

The properties and phytochemical content of FJ and FDJ were presented in Table 2. The FC of jostaberry was similar to that of blackcurrants and gooseberries, from which the hybrid originated, but the AC was richer compared to them [55].

**Table 2:** Physicochemical indicators, phytochemical content and AA of hydroethanolic extracts (70%, 1:100 ratio (*m*/*v*)) from frozen and freeze-dried jostaberry.

Indices	Samples	
	FJ	FDJ
DW, %	19.59 ± 0.06 ^a^	93.42 ±0.02 ^b^
FC, %	0.58 ± 0.01 ^a^	1.99 ± 0.02 ^b^
AC, %	0.81 ± 0.02 ^a^	3.57 ± 0.03 ^b^
TPC, mg GAE/g DW	18.67 ± 0.32 ^a^	21.37 ± 0.16 ^b^
TFC, mg RuE/g DW	4.09 ± 0.04 ^b^	3.00 ± 0.08 ^a^
TFC, mg QE/g DW	2.09 ± 0.01 ^b^	1.59 ± 0.03 ^a^
TAC, mg Cy3GE/g DW	11.24 ± 0.07 ^b^	7.00 ± 0.05 ^a^
AA by DPPH, mg TE/g DW	29.47 ± 0.22 ^b^	7.80 ±0.16 ^a^
AA by ABTS, mg TE/g DW	82.96 ± 0.56 ^b^	36.76 ± 0.34 ^a^

FJ—frozen jostaberry; FDJ—freeze-dried jostaberry; DW—dry weight; FC—fat content; AC—ash content; TPC—total polyphenol content; GAE—gallic acid equivalent; TFC—total flavonoid content; RuE—rutin equivalent; QE/g—quercetin equivalent; TAC—total anthocyanin content; Cy3GE—cyanidin-3-*O*-glucoside equivalent; AA—antioxidant activity; TE—trolox equivalent. Data are expressed as the mean of three replicates ± standard deviation (SD). Different letters indicate significant differences (*p* < 0.05).

Spectrophotometric analysis of hydroethanolic extracts (70%, 1:100 (*m*/*v*)) of FJ and FDJ revealed TPC of 18.67 and 21.37 mg GAE/g DW, respectively. Okatan recorded a TPC of 1593.92 mg GAE/100 g of fresh weight (FW) in jostaberry extract and 1223.71 mg GAE/100 g of FW in gooseberry extract [56].

Freeze-drying led to a reduction in TFC in FDJ extracts by approximately 26.7% and 24.0%, when expressed as mg RuE/g DW and mg QE/g DW, respectively. According to literature data, quercetin and rutin contents in gooseberry fruits were reported as 6.32 mg/100 g FW and 15.73 mg/100 g FW, respectively [57]. HPLC analysis showed that the highest amounts of rutin and quercetin (mg/100 g FW) were found in jostaberries (22.29 and 7.30), followed by gooseberries (15.53 and 6.16), with lower values observed in blackcurrants (15.59 and 2.74) [56].

AA determined in jostaberry extracts was increased, and the TEAC values for the DPPH• radical and the ABTS•^+^ radical cation were comparable to those reported in the literature (Table 2). Tsuda et al. reported that AA in fresh blueberry varieties, measured using the DPPH method, ranged from 52.5 to 456.2 μmol TE/g dry weight DW [58]. Okatan et al. found that fresh jostaberry extracts had ABTS values of 125.49 mg TE/100 g FW, higher than those found in gooseberries (73.23 mg TE/100 g FW) [56]. Kim et al. reported ABTS values ranging from 65.84 to 449.86 μmol TE/g DW in dry extracts of various berries [59].

In FDJ extracts, AA values determined by DPPH and ABTS were approximately 3.7 times and 2.3 times lower, respectively, compared to those in FJ extracts. This reduction could be attributed to the loss of BACs, making targets more susceptible to decomposition during fruit pretreatment. Endogenous enzymes, free radicals, transition metal ions contained in the berries, moisture, oxygen and adverse environmental conditions, as well as temperature variations during the freeze-drying process are the factors that contribute to the degradation of the compounds responsible for the AA effect: vitamin C, flavonoids, anthocyanins and others. Detailed statistical results from the ANOVA tests, including F-statistics, *p*-values, effect sizes (Cohen’s *d*), and 95% confidence intervals, are presented in Table S1.

Using both spectrophotometric and HPLC methods, the TAC in FJ extracts was more than twice as high as in FDJ extracts (Table 2 and Table 3). It was also reported by other researchers that in the process of drying berries, including freeze-drying, losses of anthocyanins are significant [32,60].

Data presented in Table 3 also indicate that freeze-drying reduced the ascorbic acid content in FDJ by nearly 45% compared to FJ. Other studies similarly reported vitamin C losses of up to 84% in freeze-dried fruits compared to their fresh counterparts [17].

Temperature fluctuations during the freeze-drying process contributed to the degradation of sensitive compounds in FDJ extracts—chlorogenic acid decreased by 8.5%, caffeic acid by 13.0%, and rutosides by 30% (Table 3). Okatan et al. reported higher chlorogenic acid content (mg/100 g FW) in fresh currants (42.56) compared to gooseberries (35.20) [56].

The increased concentrations of citric and malic acids in FDJ extracts, relative to FJ, can be attributed to the breakdown of macromolecules and the release of free acids induced by freeze-drying temperatures. According to Okatan et al., fresh jostaberries contained 14.84 mg/100 g FW of citric acid and 13.50 mg/100 g FW of malic acid [56].

Detailed statistical results from the ANOVA tests, including F-statistics, *p*-values, effect sizes (Cohen’s *d*), and 95% confidence intervals, are presented in Table S2.

### 3.2. Freeze-Dried Microparticles Preparation and Characterization

Previous studies [5] have shown that during freezing, drying or freeze-drying, vitamins, anthocyanins and other sensitive compounds in jostaberry are degraded. Consequently, in addition to crust formation, development of undesirable aromas and occurrence of browning reactions [16,33], AA is reduced in processed fruits compared to fresh fruits [5,17,32]. In addition, both fresh, frozen and processed fruits require appropriate storage conditions and facilities to maintain their biological value over long periods of time [14,15].

In order to evaluate the preservation of the biological value and antioxidant potential of BACs from jostaberry during freeze-drying and storage in the dark, under ambient conditions (22 ± 1 °C; relative humidity ≤ 75%), jostaberry extract (JE) was encapsulated in polysaccharide matrices using different combinations of biocompatible carrier agents (see Section 2), specifically maltodextrin-nutriose-pectin (MNP) and maltodextrin-nutriose-sodium alginate (MNA). The selection of combinations and ratios of biopolymers was based on preliminary studies. Maltodextrin, which is readily assimilable in the human body, was incorporated to reduce adhesion during drying and to improve the flow properties and stability of the encapsulated compounds [35]. Resistant dextrin (nutriose), sodium alginate, and pectin were chosen for their prebiotic effects and their role in improving intestinal transit [36,37]. Also, pectin and sodium alginate possess gel and film-forming capabilities, which help protect the encapsulated materials from environmental factors [33,39,40,41]. In addition, the introduction of anionic polysaccharides (sodium alginate and pectin) into the wall material could better retain and protect BACs through electrostatic interactions, which in the case of sodium alginate could be more advanced. The optimal concentration of the encapsulating agents- 30% maltodextrin, 8% nutriose, and 2% pectin or sodium alginate, produced a transparent hydrogel that retained its properties after the addition of jostaberry extract. To enhance the sustainability of the process, JE used for microencapsulation was prepared by BAC extraction using an optimal volume of 70% aqueous ethanol at a 1:6 (*w*/*v*) ratio. After ethanol evaporation, the TSS were measured using a refractometer, and the TS were determined. The contents of TPC, TAC and AA in JE were determined spectrophotometrically (Table 4).

Hydrogels were first prepared from the carrier agents and then combined with JE. The volume ratio of JE to carrier agent in the hydrogels was 1:1 (*v*/*v*), or 1:2 (*w*/*w*), if referring to JE solids versus total carrier solids. This ratio proved to be optimal, a higher concentration of solids in the josta extract gives it more acidic properties, which has been observed to decrease the solubility of sodium alginate. The resulting hydrogels formed a stable encapsulation network through physical interactions such as electrostatic forces, hydrogen bonding, and hydrophobic interactions between the biopolymer chains [18]. In the JE-loaded hydrogels (MNPJ and MNAJ), TPC and TAC were quantified, and AA was assessed using the DPPH and ABTS methods.

The microencapsulated products were freeze-dried, resulting in fine, porous MNPJ and MNAJ microparticles with sizes that can be controlled by crushing and sieving. Physicochemical properties—including MPE, MC, BD, TSS, hygroscopicity, solubility, OHC, TA, and pH—were summarized in Table 5. These physicochemical parameters were measured both at the time of production and after 12 months of storage (MNPJ_12_ and MNAJ_12_). Statistical analysis was performed by one-way ANOVA for two groups of freeze-dried microparticles (MNPJ and MNPJ_12_; MNAJ and MNAJ_12_) in order to evaluate the influence of storage time (12 months) on physicochemical indicators. Detailed statistical results from the ANOVA tests, including F-statistics, *p*-values, effect sizes (Cohen’s *d*), and 95% confidence intervals, are presented in Table S3.

The MPE was 87.7% for MNPJ and for MNAJ these values are higher, at 88.9%. The MPE could potentially be higher with improved handling to minimize product losses during manufacturing. Da Rosa et al. reported an encapsulation efficiency of 96.80% for blueberry extract using a maltodextrin/corn starch/inulin blend, and 98.83% when gum Arabic was also included [24].

MC is a critical quality parameter that influences freeze-drying efficiency, microparticle stability, flowability, adhesion, degradation of BACs, and the risk of microbial growth. The MC values for MNPJ (2.61%) and MNAJ (2.80%) were within the acceptable range reported in the literature and showed only a slight increase with a significant difference (*p* < 0.03) after one year of storage. Typically, dry food powders require an MC between 3% and 10% to remain stable during storage [61]. Dag et al. reported moisture contents of 9–11% in freeze-dried maltodextrin-gum arabic-goldenberry juice powders [62].

The bulk density (BD) of the microparticles increased towards the end of the storage period, with significant differences observed between the values measured in the MNPJ and MNPJ_12_ groups, as well as in MNAJ and MNAJ_12_, at production and after 12 months (*p* < 0.00002), Table S3, probably due to the increase in moisture content. A BD below 0.57 g/cm^3^ is often associated with air ingress into the product, which can promote oxidation during storage [63]. Franceschinis et al. [64] reported an even lower BD (0.45 g/cm^3^) for lyophilized blackberry powder encapsulated with maltodextrin.

TSS, hygroscopicity and solubility are largely determined by the solubility characteristics and the presence of hydrophilic groups in the carrier agents. Pectin and sodium alginate dissolve more slowly than maltodextrin, which explains the slightly lower TSS values observed for MNPJ and MNAJ (Table 5) compared to microparticles prepared with maltodextrin alone (11.56 °Bx) [65]. Towards the end of the 12 months, the TSS values of the microparticles in both variants decreased slightly, with significant differences (*p* < 0.015). It is well established that powders containing higher amounts of maltodextrin dissolve more readily in water, with reported solubility typically exceeding 90%. Solubility is also influenced by particle size—the smaller the particles, the greater the surface area in contact with the solvent [64,65]. The solubility of MNPJ microparticles (55.55%) was higher than that of MNAJ microparticles (39.40%). After storage, the values of this indicator decreased, but significant differences were found between the values determined in the MNPJ and MNPJ_12_ samples (*p* = 0.0002). The reduced solubility of pectin and sodium alginate is a characteristic property of macromolecular compounds, whose dissolution is preceded by a swelling phase. These properties contribute to better protection of the encapsulated compounds. Hygroscopicity indicates the potential of microparticles to absorb moisture relative to ambient humidity. Powders can be classified based on their hygroscopicity as non-hygroscopic (<10%), slightly hygroscopic (10–15%), or hygroscopic (15–20%) [66]. For comparison, microparticles containing anthocyanins from *Hibiscus sabdariffa* L. encapsulated in maltodextrin showed a moisture content of 5.28% and hygroscopicity of 17.50% [67]. The hygroscopicity of MNPJ and MNPJ_12_ did not change significantly after the end of the storage period. Significant differences were found between the values determined in the MNAJ samples at the date of production and after 12 months of storage (*p* < 0.003), probably due to the higher hygroscopic nature of sodium alginate, similar to other sodium salts. OHC depends largely on the structure of the encapsulation matrix as well as on the nature of the encapsulated product, including the polarity of the molecules and the types of functional groups present, which can impart hydrophilic or lipophilic properties to the microcapsules [43]. The OHC of MNAJ microparticles containing sodium alginate (1.48%) was lower than that of MNPJ microparticles (3.44%), likely due to reduced accessibility of hydrophobic components on their surface. After storage, OHC decreased in both types of microparticles. Significant differences were found between the OHC values determined in the samples at the date of production and after 12 months of storage (*p* < 0.0007) due to the increase in MC in the samples. TA was higher in MNPJ microparticles due to the greater number of free carboxyl groups in pectin compared to MNAJ, which contained sodium alginate (Table 5). TA measured at the end of the storage period was significantly lower in both formulations compared to the values at production (*p* < 0.007). Consequently, pH values increased in both formulations with significant differences (*p* < 0.0006) being recorded before and after storage.

The differences between the FC values, determined in microparticles before and after storage, as well as the AC values, were not statistically significant across all variants (Table S3). After 12 months of storage in the dark under ambient conditions, the physicochemical properties of the microparticles remained largely stable. One-way ANOVA analysis revealed a slight increase in MC, BD and pH, accompanied by a decrease in OHC, hygroscopicity, solubility and TA at the end of the storage period. Significant differences (*p* < 0.05) were observed between values measured at production and after 12 months of storage.

### 3.3. Color Analysis

Color is a key chromatic parameter that significantly influences consumer choice and purchasing decisions. Therefore, the color of a food product must be attractive and remain stable throughout its shelf life, even when stored in transparent packaging. Images of the obtained microparticles were shown in Figure S1. The color parameters of the analyzed samples were presented in Table 6.

The results indicated that the MNAJ sample exhibited higher lightness (L*) compared to MNPJ, with values of 46.85 and 38.74, respectively—an increase of approximately 21%. This difference in lightness may be perceptible to the naked eye, as shown in Figure S1. Nthimole et al. reported a maximum increase in lightness of 18% between encapsulated powders using gum agar, maltodextrin, and waxy starch, with the highest value observed in powders where waxy starch was used as the wall material, followed by maltodextrin [65].

The red-green (a*) values were positive across all samples, reflecting the characteristic red-to-purple color of jostaberry. These values were high for all samples, indicating that the freeze-drying process effectively preserved the product’s color. The low pH values also contributed significantly to the enhancement of red hues. Accordingly, the MNPJ sample exhibited the most intense red color (a* = 34.98), corresponding with its lowest pH and highest TA.

The yellow-blue (b*) values were negative, indicating the presence of blue tones, which are also characteristic of jostaberry. A more intense blue hue was observed in the MNAJ sample. The hue angle (h*) showed minimal variation between samples—95.17 for MNPJ and 94.10 for MNAJ—confirming that red hues predominated. The chroma (C*) values of the encapsulated powders remained relatively unchanged, at 7.53 for MNPJ and 6.66 for MNAJ, indicating that both samples maintained a saturated, vivid color.

After 12 months of storage, the color of MNPJ_12_ and MNAJ_12_ microparticles remained largely stable (Figure S1). After storing the samples for 12 months, the same trend of increasing L* was visible (*p* < 0.014). The a* decreased for both types of microcapsules (*p* < 0.0001). The b* became more intense after storage, and the values for C* and h* changed slightly (*p* < 0.002). Any visible color differences in the images were likely due to variations in photographic conditions rather than actual degradation. Detailed statistical results from the ANOVA tests, including F-statistics, *p*-values, effect sizes (Cohen’s *d*), and 95% confidence intervals, are presented in Table S4.

Additionally, the color stability of microparticle aqueous solutions was evaluated as a function of pH. It was found that the red color of MNPJ and MNAJ solutions remained stable in the pH range of 2.0 to 5.5 (Figure S2). Therefore, the incorporation of these microparticles into mildly acidic food products can impart a stable red color.

### 3.4. Biological Value and Antioxidant Potential of Microparticles

Polyphenols, pigments, and vitamins found in fruits, vegetables, and plants are largely responsible for their antioxidant activity. However, these compounds are inherently unstable and prone to degradation during processing, exposure to adverse conditions, and storage. Encapsulation offers an effective strategy to preserve their biological activity over prolonged periods.

The mechanistic aspects of the encapsulation process of JE involve multiple types of interactions between the combined biopolymer matrix (maltodextrin, resistant dextrin (nutriose) and anionic polysaccharides) and the encapsulated compounds: phenolic acids, flavonoids, pigments, vitamins and others. A greater diversity in the electrophilic or nucleophilic character of the functional groups in the encapsulated materials implies more advanced physicochemical interactions, which increase the stability of BACs, preserving their color, AA, biological value during processing and storage for longer periods [24,25,26,27].

In the elaborated microparticles, BACs are retained and stabilized through physicochemical interactions, which include the following:

(a) Hydrogen bonds between the hydroxyl (–OH) and carboxyl (–COOH) groups of polyphenols (e.g., caffeic and chlorogenic acids), the hydroxyl groups of anthocyanins, and the hydroxyl groups of polysaccharides, such as maltodextrin and resistant dextrin, as well as the carboxyl groups of anionic polysaccharides, such as pectin and sodium alginate.
(b) Electrostatic interactions, especially between the protonated forms of anthocyanins in acidic media, which may be stronger with sodium alginate (–COONa), thus enhancing retention and protection under acidic conditions.
(c) Hydrophobic interactions, where hydrophobic regions of bioactive compounds associate with less polar regions of the wall encapsulating materials, such as resistant dextrin.

In addition to these chemical interactions, BACs are physically trapped in the polysaccharide network formed during the encapsulation and freeze-drying processes. This physical encapsulation further protects them from light, heat, oxygen, and the degrading enzymes typically present in the plant matrix [68,69].

#### 3.4.1. Total Phenolic, Total Anthocyanin Content and Antioxidant Activity

The TPC, TAC, and AA of the encapsulated materials were measured before and after freeze-drying (Table 7). The TPC (5.02 and 4.67 mg GAE/g DW) and TAC (2.57 and 2.37 mg Cy3GE/g DW) in MNPJ and MNAJ hydrogels (prior to freeze-drying) were nearly halved compared to the values in the JE used for encapsulation (Table 4), due to dilution effects. After freeze-drying, these values decreased slightly, with significant differences (*p* < 0.0001), indicating that BACs were retained in both types of encapsulating agent. AA values before freeze-drying versus after were significantly different (*p* < 0.0001) and showed a moderate decrease after processing (Table S5).

Casati et al. reported TPC values between 769 and 1472 mg GAE/100 g DW in blueberry, elderberry, blackcurrant, and Maqui berry freeze-dried powders developed from a mixture of maltodextrin and gum Arabic [70].

A comparison of TAC values in FJ and FDJ extracts (Table 2 and Table 3) revealed that the freeze-drying process reduced the anthocyanin concentration in FDJ by more than two-fold. In contrast, when anthocyanins were encapsulated before freeze-drying, the TAC reduction was limited to approximately 1.1-fold (Table 7), indicating that encapsulation mitigated anthocyanin losses during the drying process.

Bakowska-Barczak et al. reported TPC values in spray-dried microparticles encapsulated with maltodextrin (MD) of different dextrose equivalents (DE)—1251.4, 1243.0, and 1163.0 mg GAE/100 g for MD 11, MD 18, and MD 21, respectively. TAC values were 452.8, 451.3, and 430.4 mg/100 g, respectively [28].

In another study, the TAC of *Hibiscus sabdariffa* L. extract microparticles encapsulated in maltodextrin was 425 mg/100 g, and in yeast hulls, it reached 515 mg/100 g [67].

Literature data also show that the anthocyanin-to-wall-material ratio significantly affects the anthocyanin content in microcapsules. Mazuco et al. reported a TAC of 116.89 mg/100 g and an encapsulation efficiency of 20.45% at an anthocyanin-to-polymer ratio of 2:1. When the ratio was increased to 2:3, TAC rose to 151.68 mg/100 g, and encapsulation efficiency increased to 21.11% [71].

AA values determined in MNPJ and MNAJ freeze-dried microparticles by the DPPH method were 5.69 and 6.06 mg TE/g DW, respectively, and by the ABTS method, 11.35 and 13.76 mg TE/g DW, respectively. These results suggested that the type of biopolymer matrix (pectin or sodium alginate) significantly (*p* < 0.05) affected the AA of the microparticles before and after lyophilization. Bakowska-Barczak and Kolodziejczyk found DPPH values of 4.5, 4.1, and 4.2 mM TE/100 g for black currant microparticles spray-dried at 150 °C using maltodextrin with DE 11, 18, and 21, respectively. ABTS values for the same samples were 9.3, 8.9, and 9.1 mM TE/100 g [28].

Research indicates that jostaberry phytochemicals encapsulated in a combined biopolymer matrix were effectively protected against degradation and oxidation during the freeze-drying. Comparative analysis of the data presented in Table 2 and Table 3 revealed that the freeze-drying process of FJ substantially reduced AA, with DPPH values decreasing by 3.7-fold and ABTS values decreasing by 2.3-fold in FDJ. In contrast, freeze-drying of bioactive compounds in encapsulated form resulted in significantly lower losses: AA decreased by approximately 1.1-fold (DPPH) and 1.5-fold (ABTS) for both types of microparticles (Table 7).

Detailed statistical results from the ANOVA tests, including F-statistics, *p*-values, effect sizes (Cohen’s *d*), and 95% confidence intervals, are presented in Table S5.

#### 3.4.2. Retention Efficiency and Storage Stability of Microencapsulated Phytochemicals from Jostaberry

RE of jostaberry phytochemicals in the biopolymer matrix of freeze-dried microparticles was calculated at the time of production and every three months during storage, alongside determination of TPC and TAC (Table 8). The summaries of the ANOVA and Tukey post hoc test results for the biological and antioxidant potential of MNPJ and MNAJ microparticles during storage are presented in Tables S6 and S7. All measured variables have statistically significant ANOVA *p* values (<0.05), indicating that at least one mean at one time point differs from the others. Tukey’s post hoc HSD analysis revealed multiple significant differences between storage intervals for all measured parameters. While DPPH and ABTS values degrade constantly, others (TPC, TAC, RE) show sudden changes after specific storage intervals.

TPC showed a slight increase during the first three months of storage in both samples. The significant difference between 3 months versus 12 months of storage (MNPJ, *p* = 0.037 and MNAJ, *p* =0.020) indicating an increase in TPC in both types of microparticles up to 3 months (Tables S6 and S7). Other differences were not significant, suggesting that most losses occurred between the intermediate and final stages. After 12 months, TPC decreased by 8.0% in MNPJ, while no reduction was observed in MNAJ, compared to the values reported after manufacture. The initial RE of polyphenols at the time of production was 93.1% for MNPJ and 90.0% for MNAJ. After 12 months of storage, these values were 85.5% and 90.7%, respectively (Table 8). For MNPJ, almost all pairwise comparisons of RE TPC were statistically significant (*p* < 0.003), except for the 6-month period versus the date of production (*p* = 0.21) (Table S6). These results indicate that the RE of polyphenols in MNPJ peaked at 3 months, followed by a sharp decline at 6 months and a further reduction at 12 months, reaching levels below those measured at production.

In MNAJ, only the comparison between 3 months and the date of production was statistically significant (*p* = 0.0227), while all other differences were not significant (Table S7). RE values in this formulation peaked after 3 months of storage, then decreased slightly after 12 months, returning to levels similar to those at production. These findings suggest that polyphenols are better protected during storage in sodium alginate-containing microparticles. The TAC decreased slightly over the storage period—by approximately 12.2% in MNPJ and only 2.8% in MNAJ. Significant differences for TAC values at 12 versus 6 months, in both types of microparticles (*p* < 0.002), indicate small but consistent decreases after prolonged storage. Similar findings were reported by other researchers, who observed that TAC in powders encapsulated with yeast hulls and maltodextrin decreased over a 10-week storage period from 99% to 75% and 88%, respectively, as storage temperature increased from 5 °C to 37 °C [67]. Da Rosa et al. [24] found that blueberry extract encapsulated in DE20 maltodextrin, hi-maize resistant starch, inulin and gum arabic, followed by spray-drying, had the lowest anthocyanin losses under all storage conditions: 5.94% at room temperature, 5.82% at refrigeration and 4.45% at freezing temperature, with a half-life of: 679.81, 693.32 and 913.41 days, respectively, under the mentioned conditions. The RE of anthocyanins had values of 95.7% and 93.4%, respectively, at the time of production for MNPJ and MNAJ microparticles (Table 8), demonstrating that these compounds in encapsulated form are better protected from degradation during the lyophilization process. The RE of TAC showed multiple significant changes, especially between 3 and 6 months and at 12 months. In MNPJ almost all pairwise differences were significant (*p* < 0.002), except for 3 months versus 6 months (*p* = 0.182), demonstrating a high sensitivity to storage changes (Table S6). These values in MNAJ only at 3 months versus after production were significantly different (*p* = 0.0227), all other differences were insignificant (Table S7). The data analysis indicates that anthocyanins are better protected in MNAJ microparticles; in MNPJ, we observe a pronounced decrease in TAC and retention, starting with the 6th month of storage.

Other studies have shown that the retention of TPC and TAC in spray-dried blackberry juice powder with maltodextrin was 73% and 75%, respectively [45]. Similarly, Yu and Lv observed retention efficiencies of 91.44% for TAC and 95.12% for TPC in freeze-dried microcapsules [72].

As a result of the data analysis, it is concluded that the BACs encapsulated in MNAJ microparticles, which contained sodium alginate, were better protected during 12 months of storage. This can be explained by alginate’s ability to form tougher films, which are protective barriers against abiotic factors, as well as through the electrostatic interactions between the flavylium cations (the protonated form of anthocyanins in acidic environments) and the carboxyl groups from sodium alginate. Statistical analysis revealed significant changes in the DPPH and ABTS antioxidant profiles of the microparticles during the storage period. Antioxidant activity (AA) showed the greatest sensitivity to time, with free radical scavenging capacity decreasing significantly between 12 months and earlier stages (*p* < 0.0001). For both MNPJ and MNAJ, DPPH and ABTS values differed significantly between 12 and 3 months (*p* < 0.003), 12 and 6 months (*p* < 0.015), and between 12 months and the date of manufacture (*p* < 0.0004) (Tables S6 and S7). These findings indicate that most of the AA changes occur during later stages of storage, with a gradual decrease observed for both types of microparticles.

Bakowska-Barczak et al. demonstrated that the AA of polyphenols encapsulated in maltodextrin remained unchanged after 12 months of storage at 8 °C, and slightly decreased when stored at 25 °C [28]. Similarly, polyphenol extracts from cloudberry (*Rubus chamaemorus*) stabilized by freeze-drying with two types of maltodextrin (DE 5–8 and DE 18.5) effectively protected polyphenols from oxidation, maintaining their antioxidant activity during storage [73].

### 3.5. Microstructure of Microparticles

**Figure 1:**
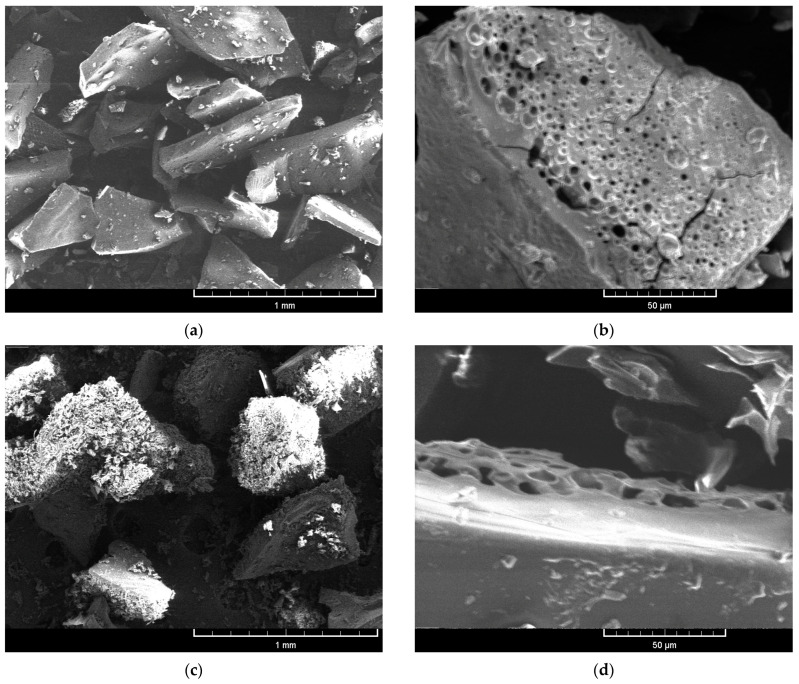
Scanning electron microscope images of microparticles: (**a**,**b**) jostaberry extract encapsulated in a maltodextrin–nutriose–pectin matrix; (**c**,**d**) jostaberry extract encapsulated in a maltodextrin–nutriose–sodium alginate matrix. Images captured at 80× magnification (**a**,**c**) and 1000× magnification (**b**,**d**), all at 10.0 kV accelerating voltage.

The microstructure and surface morphology of MNPJ and MNAJ microparticles were investigated by SEM. The results are presented in Figure 1.

The median diameter of the average value of the particle size distribution D50 was 320 ± 15 and 360 ± 20 µm for MNPJ and MNAJ, respectively. The shapes were irregular, and the superstructure was predominantly amorphous, with denser crystalline regions observed in MNAJ (Figure 1c,d). No areas of conglomeration were detected, indicating that the microparticles are non-adhesive. Some surfaces appear rough and porous, resulting from the sublimation of ice crystals during freeze-drying. It is known that rough and porous surfaces are more susceptible to oxidation than smooth ones, due to the larger areas exposed to air. [74]. Ideally, microcapsules should be spherical, smooth, and uniform, without cracks or collapses on their walls [75].

Nevertheless, the microencapsulated compounds from jostaberry in the developed polysaccharide combinations were effectively protected from exposure to adverse conditions during freeze-drying and storage for a period of 12 months.

### 3.6. Relationship Between Physicochemical Characteristics, pH and Color Parameters

**Figure 2:**
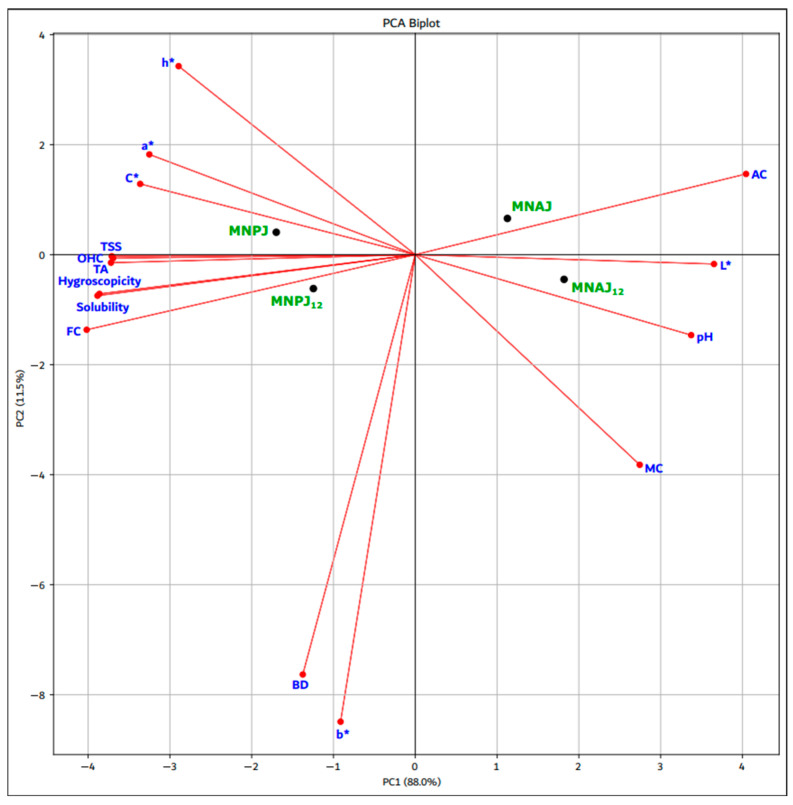
Principal component analysis: MNAJ—josta extract in maltodextrin-nutriose-sodium alginate matrix; MNPJ—josta extract in maltodextrin-nutriose-pectin matrix; MNPJ_12_ and MNAJ_12_—microparticles after 12 months of storage; FC—fat content; TA—titratable acidity, OHC—oil holding capacity; TSS—total soluble solids; AC—ash content; MC—moisture content; BD—bulk density; L*—lightness; a*—red-green parameter; b*—yellow-blue parameter; C*— chromaticity, h^*^—hue angle.

PCA was used to illustrate the relationship between physicochemical characteristics, pH and color parameters determined in MNAJ and MNAJ_12_, MNPJ and MNPJ_12_, Figure 2 .

The first two principal components, PC1 and PC2, accounted for 88.0% and 11.5% of the overall variance. highlights that some parameters are closely associated, showing highly significant correlations (*p* < 0.05), such as: TSS and hygroscopicity, solubility, OHC, TA, FC (r = 1.00); h* and a* (r = 0.97), C* (r = 0.94); pH and L* (r = 0.98), AC (r = 0.94), MC (r = 0.92), BD and b* (r = 0.99), Figure S3. PC1 was closely associated with TSS, OHC, TA, hygroscopicity, solubility, FC, C*, a*, L*, pH, AC, whereas PC2 was closely associated with BD, b*, MC and h*. According to the PCA plot, depending on the biopolymer matrix and the microparticle storage time, the closest to each other are: MNPJ and MNPJ_12_, which are located in the center on the left of the plot, MNAJ and MNAJ_12_ are also positioned in the center, but on the right of the PCA. Both PCA components distinguish MNPJ and MNPJ_12_ from MNAJ and MNAJ_12_, indicating an inverse correlation between microcapsules having different biopolymer matrix. The first component PC1 distinguishes MNPJ, MNPJ_12_ from MNAJ, MNAJ_12_, while the second component PC2 distinguishes MNPJ, MNAJ from microcapsules MNPJ_12_, MNAJ_12_. Regarding the correlations of microcapsules with the analyzed characteristics, it seems that MNPJ and MNPJ_12_ are rather related to hygroscopicity, solubility, FC, TA, OHC, TSS, MC, a*, C*, h*; MNAJ and MNAJ_12_ are related to pH, MC, L* and AC. b* and BD are removed from all analyzed samples.

## 4. Limitations of the Study

Microencapsulation of plant extracts is an alternative for the valorization of fruits, vegetables and medicinal plants, as well as for preserving the antioxidant potential of BACs for longer periods. At the same time, further research on the release mechanisms is needed to adapt the microencapsulation process to different food applications.

A limitation of this study is the lack of control of some abiotic factors, the short storage period analyzed, which may not fully reflect the long-term stability of the encapsulated compounds. Further research is needed to optimize the efficiency of encapsulation of bioactive compounds from jostaberry in polysaccharide matrices. It is also important to study in depth the approach from a mechanistic perspective that explains the biochemical, physical or molecular processes through which the phenomena related to the incorporation of BACs into biopolymers, the bioavailability of microparticles and their behavior in the digestion phases occur.

Also, the difficulties associated with scaling up processes from laboratory to industrial production cannot be overlooked, due to the need for specialized equipment. Added to this are the cost of encapsulation materials and energy costs. These aspects generate higher prices for the final product and the risk of it becoming less affordable for some consumers.

## 5. Conclusions

It was concluded that encapsulation of jostaberry extract in a polysaccharide matrix, followed by lyophilization, effectively preserved its biological value and antioxidant potential, both during processing and during 12 months of storage, while improving handling properties. The encapsulated phytochemicals showed substantially less degradation during lyophilization compared to the unencapsulated ones in FDJ. AA determined in MNPJ and MNAJ microparticles by DPPH and ABTS assays decreased only 1.1- and 1.5-fold, respectively, while in FDJ, AA decreased 3.7-fold (DPPH) and 2.3-fold (ABTS), respectively.

Tukey’s post hoc HSD analysis revealed multiple significant differences between storage intervals (3, 6 and 12 months) for all measured parameters. A gradual decrease in DPPH and ABTS values was observed, which became more accelerated after the first three months of storage. TPC, TAC, and their RE showed changes after specific storage intervals.

After 12 months of storage, TPC and TAC decreased by 8.2% and 12.2% in MNPJ microparticles and by 3.3% and 3.9% in MNAJ, respectively. After 12 months, AA determined in MNPJ decreased by 8.4% (DPPH) and 2.7% (ABTS), and in MNAJ, it decreased by 5.4% and 3.7%, respectively. As a result of data analysis, it was concluded that BACs encapsulated in MNAJ microparticles, which contained sodium alginate, were better protected during storage.

After 12 months of storage in the dark under ambient conditions, the color and physicochemical properties of the microparticles remained largely stable. One-way ANOVA analysis (*p* < 0.05) revealed a slight increase in MC, BD and pH, accompanied by a decrease in OHC, hygroscopicity, solubility, and TA at the end of the storage period.

The microencapsulation of jostaberry phytochemicals represents an effective strategy for preserving the antioxidant potential of BACs during processing and storage. Furthermore, the developed microcapsules warrant investigation for potential applications in the formulation of dietary supplements and functional foods.

## 6. Future Outlook

Future research should focus on evaluating the long-term stability of encapsulated compounds under various storage conditions, as well as on the release mechanisms of BACs in simulated gastrointestinal environments. Scaling up the encapsulation process and integrating it into real food matrices will be essential for industrial applications, especially since seasonal fruits are perishable and require specific pretreatment, storage space, and conditions to maintain their biological value.

Exploring new combinations of biopolymers with different plant extracts, including those from plant waste, can enhance both the sustainability of the process and the health benefits for consumers. These developments contribute to the production of supplements and functional foods with enhanced bioavailability and nutritional value, improved color and texture, and extended shelf life.

## Figures and Tables

**Table 3:** Quantification and profiling of biologically active compounds in FJ and FDJ 70% hydroethanolic extracts (1:100, *m*/*v*) using HPLC and capillary electrophoresis.

Biological Active Compounds	Samples	
	FJ	FDJ
Anthocyanins, Cy3-*O*-Glc, mg/g DW	14.00 ± 0.02 ^b^	5.95 ± 0.03 ^a^
Ascorbic acid, mg/g DW	3.60 ± 0.02 ^b^	1.99 ± 0.01 ^a^
Chlorogenic acid, mg/g DW	2.00 ± 0.02 ^b^	1.83 ±0.01 ^a^
Caffeic acid, mg/g DW	0.31 ± 0.01 ^b^	0.27 ± 0.01 ^a^
Rutoside, mg/g DW	1.36 ± 0.02 ^b^	0.95 ±0.02 ^a^
Malic acid, mg/g DW	3.43 ± 0.02 ^a^	4.11 ± 0.03 ^b^
Citric acid, mg/g DW	3.21 ± 0.01 ^a^	3.94 ± 0.02 ^b^
Fumaric acid, mg/g DW	5.02 ± 0.03 ^a^	7.15 ± 0.03 ^b^

FJ—frozen jostaberry; FDJ—freeze-dried jostaberry, Cy3-*O*-Glc—cyanidin-3-*O*-glucoside. Data are expressed as the mean of three replicates ± standard deviation (SD). Different letters indicate significant differences (*p* < 0.05).

**Table 4:** Jostaberry Extracts Characteristics.

Indices	JE
TSS, °Bx	18.12 ± 0.20
TS, %	19.95 ± 0.40
TPC, mg GAE/g DW	13.55 ± 1.23
TAC, mg Cy3GE/g DW	7.15 ± 0.13
AA by DPPH, mg TE/g DW	14.84 ± 0.33
AA by ABTS, mg TE/g DW	40.79 ± 2.37

The JE for encapsulation was obtained with a 70% hydroethanolic solution, in a ratio of 1:6 (*m*/*v*), subsequently, the ethanol was evaporated in vacuum. TSS—total soluble solids; °Bx—Brix; TS—total solids; TPC—total polyphenol content; AA—antioxidant activity; GAE—gallic acid equivalent; DW—dry weight; TAC—total antocyanin content; Cy3GE—cyanidin-3-*O*-glucoside equivalent; TE—trolox equivalent. Data are expressed as the mean of three replicates ± standard deviation (SD).

**Table 5:** Physicochemical indicators of freeze-dried microparticles.

Physicochemical Indicators	Microparticles			
	MNPJ	MNPJ_12_	MNAJ	MNAJ_12_
MPE, %	87.74 ± 0.05	nd	88.90 ±0.01	nd
MC, %	2.61 ± 0.01 ^a^	2.76 ± 0.01 ^b^	2.80 ± 0.01 ^a^	2.93 ± 0.01 ^b^
BD, g/cm^3^	0.781 ± 0.001 ^a^	0.820 ± 0.001 ^b^	0.752 ± 0.001 ^a^	0.790 ± 0.001 ^b^
TSS, °Bx	9.22 ± 0.01 ^b^	9.13 ± 0.03 ^a^	8.47 ± 0.02 ^b^	8.25 ± 0.03 ^a^
Hygroscopicity, %	7.62 ± 0.01 ^a^	7.61 ± 0.02 ^a^	7.43 ± 0.01 ^b^	7.39 ± 0.01 ^a^
Solubility, %	55.55 ± 0.06 ^b^	53.67 ± 0.09 ^a^	39.40 ± 0.02 ^b^	37.12 ± 0.05 ^a^
OHC, %	3.44 ± 0.06 ^b^	3.03 ± 0.03 ^a^	1.48 ± 0.05 ^b^	1.16 ± 0.03 ^a^
TA, %	16.02 ± 0.17 ^b^	15.45 ± 0.10 ^a^	12.88 ± 0.08 ^b^	12.31 ± 0.11 ^a^
pH	3.28 ± 0.01 ^a^	3.36 ± 0.01 ^b^	3.72 ± 0.01 ^a^	4.04 ± 0.01 ^b^
FC, %	0.07 ± 0.01 ^a^	0.07 ± 0.01 ^a^	0.05 ± 0.01 ^a^	0.05 ± 0.01 ^a^
AC, %	0.46 ± 0.02 ^b^	0.44 ± 0.04 ^a^	1.06 ± 0.03 ^b^	1.02 ± 0.02 ^a^

MNPJ—josta extract in maltodextrin-nutriose-pectin matrix; MNAJ—josta extract in maltodextrin-nutriose-sodium alginate matrix. MNPJ_12_ and MNAJ_12_—microparticles after 12 months of storage. MPE—microparticles production efficiency; MC—moisture content; BD—bulk density; TSS—total soluble solids; OHC—oil holding capacity; TA—titratable acidity; FC—fat content; AC—ash content. Data are expressed as the mean of three replicates ± standard deviation (SD); nd—not determined. Different letters indicate significant differences (*p* < 0.05).

**Table 6:** CIELab color parameters of freeze-dried microparticles.

Microparticles	CIELab Color Parameters				
	L*	a*	b*	C*	h*,°
MNPJ	38.74 ± 0.24 ^a^	34.98 ± 0.21 ^b^	−4.82 ± 0.08 ^b^	7.53 ± 0.04 ^b^	35.17 ± 0.09 ^b^
MNPJ_12_	40.21 ± 0.56 ^b^	32.88 ± 0.11 ^a^	−3.56 ± 0.02 ^a^	7.21 ± 0.03 ^a^	34.57 ± 0.08 ^a^
MNAJ	46.85 ± 0.29 ^a^	29.46 ± 0.09 ^b^	−5.47 ± 0.06 ^b^	6.66 ± 0.05 ^b^	34.10 ± 0.01 ^b^
MNAJ_12_	48.96 ± 0.14 ^b^	26.73 ±0.13 ^a^	−4.22 ± 0.01 ^a^	6.40 ± 0.04 ^a^	32.74 ± 0.06 ^a^

MNPJ—josta extract in maltodextrin-nutriose-pectin matrix; MNAJ—josta extract in maltodextrin-nutriose-sodium alginate matrix; MNPJ_12_ and MNAJ_12_—microparticles after 12 months. L* denotes lightness; a* corresponds to the red-green axis; b* to the yellow-blue axis; C* represents chromaticity; h* indicates the hue angle. Values are expressed as mean ± standard deviation (SD) from three replicates. Different letters denote statistically significant differences at *p* < 0.05.

**Table 7:** TPC, TPA and AA in encapsulated materials before and after freeze-drying.

Parameters	MNPJ		MNAJ	
	Before Freeze-Drying	After Freeze-Drying	Before Freeze-Drying	After Freeze-Drying
TPC, mg GAE/g DW	5.02 ± 0.01 ^b^	4.67 ± 0.01 ^a^	4.57 ± 0.02 ^b^	4.07 ± 0.03 ^a^
TAC, mg Cy3GE/g DW	2.57 ± 0.02 ^b^	2.37 ± 0.01 ^a^	2.33 ± 0.01 ^b^	2.17 ± 0.0 ^a^
AA by DPPH, mg TE/g DW	6.32 ± 0.01 ^b^	5.69 ± 0.03 ^a^	6.49 ± 0.02 ^b^	6.06 ±0.02 ^a^
AA by ABTS, mg TE/g DW	17.89 ± 0.05 ^b^	11.35 ± 0.02 ^a^	18.92 ± 0.07 ^b^	13.76 ± 0.06 ^a^

MNPJ—microparticles of josta extract in maltodextrin-nutrose-pectin matrix; MNAJ—josta extract in maltodextrin-nutrose-sodium alginate matrix. TPC—total polyphenol content; GAE—gallic acid equivalent; DW—dry weight; TAC—total anthocyanin content; Cy3GE—cyanidin-3-*O*-glucoside equivalent; AA—antioxidant activity; TE—trolox equivalent. Values are expressed as mean ± standard deviation (SD) from three replicates. Different letters denote statistically significant differences at *p* < 0.05.

**Table 8:** Biological value and antioxidant potential of microparticles during storage.

Indices	MNPJ				MNAJ			
	After Production	After 3 Months	After 6 Months	After 12 Months	After Production	After 3 Months	After 6 Months	After 12 Months
TPC, mg GAE/g DW	4.67 ± 0.02 ^b^	4.84 ± 0.03 ^c^	4.61 ± 0.01 ^b^	4.30 ± 0.0 ^a^	4.07 ± 0.02 ^a^	4.29 ± 0.01 ^b^	4.25 ± 0.02 ^b^	4.14 ± 0.01 ^a,b^
TPC RE, %	93.1 ± 0.1 ^b^	96.3 ± 0.1 ^c^	91.8 ± 0.2 ^b^	85.5 ± 0.0 ^a^	90.0 ± 0.2 ^a^	93.8 ± 0.4 ^c^	92.9 ± 0.3 ^b^	90.7 ± 0.2 ^a,b^
TAC, mg Cy3GE/g DW	2.46 ± 0.01 ^b^	2.39 ± 0.01 ^b^	2.39 ± 0.01 ^b^	2.16 ± 0.02 ^a^	2.17 ± 0.01 ^a^	2.16 ± 0.01 ^a^	2.30 ± 0.0 ^b^	2.11 ± 0.01 ^a^
TAC RE, %	95.7 ± 0.1 ^c^	92.9 ± 0.2 ^b^	91.4 ± 0.2 ^b^	84.0 ± 0.1 ^a^	93.4 ± 0.1 ^b^	92.9 ± 0.1 ^b^	89.3 ± 0.1 ^a^	89.8 ± 0.1 ^a^
AA by DPPH, mg TE/g DW	5.60 ± 0.02 ^c^	5.61 ± 0.01 ^c^	5.49 ± 0.02 ^b^	5.13 ± 0.01 ^a^	6.10 ± 0.03 ^b^	6.23 ± 0.02 ^c^	6.20 ± 0.02 ^c^	5.77 ± 0.01 ^a^
AA by ABTS, mg TE/g DW	11.02 ± 0.05 ^a,b^	11.20 ± 0.06 ^b^	11.11 ± 0.05 ^b^	10.72 ± 0.04 ^a^	13.37 ± 0.06 ^a,b^	13.42 ± 0.08 ^b^	13.25 ± 0.04 ^a,b^	12.87 ± 0.06 ^a^

MNPJ—josta extract in maltodextrin-nutrose-pectin matrix; MNAJ—josta extract in maltodextrin-nutrose-sodium alginate matrix. TPC—total polyphenol content; GAE—gallic acid equivalent; DW—dry weight; RE—retention efficiency; TAC—total anthocyanin content; Cy3GE—cyanidin-3-*O*-glucoside equivalent; AA—antioxidant activity; TE—trolox equivalent . Values are expressed as mean ± standard deviation (SD) from three replicates. Different letters (a–c) denote statistically significant differences at *p* < 0.05.

## Data Availability

The original contributions presented in the study are included in the article; further inquiries can be directed to the corresponding authors.

## Supplementary Materials

The following supporting information can be downloaded at: https://www.mdpi.com/article/10.3390/foods14173092/s1, Figure S1: Photos of freeze-dried microparticles; Figure S2: Color change of aqueous solutions of microparticles as a function of pH; Figure S3: The correlation values between physicochemical characteristics, pH and color parameters in microparticles. FC—fat content; TA—titratable acidity, OHC—oil holding capacity; TSS—total soluble solids; AC—ash content; MC—moisture content; BD—bulk density; L*—lightness; a*—red-green parameter; b*—yellow-blue parameter; C*—chromaticity, h*—hue angle of MNAJ—josta extract in maltodextrin-nutriose-sodium alginate matrix; MNPJ—josta extract in maltodextrin-nutriose-pectin matrix; MNPJ_12_ and MNAJ_12_—microparticles after 12 months of storage. Table S1: Summary of ANOVA results including F-statistics, *p*-values, Cohen’s *d* effect sizes, and 95% confidence intervals for each comparison for physicochemical indicators, phytochemical content and AA of hydroethanolic extracts (70%, 1:100 ratio (*m*/*v*)) from frozen and freeze-dried jostaberry; Table S2: Summary of ANOVA results including F-statistics, *p*-values, Cohen’s *d* effect sizes, and 95% confidence intervals for each comparison for biological active compounds identified and quantified by the HPLC and capillary electrophoresis method in jostaberry hydroethanolic 70% extract (1:100, *m*/*v*) from frozen and freeze-dried jostaberry; Table S3: Summary of ANOVA results including F-statistics, *p*-values, Cohen’s *d* effect sizes, and 95% confidence intervals for each comparison for physicochemical indicators of freeze-dried microparticles; Table S4: Summary of ANOVA results including F-statistics, *p*-values, Cohen’s *d* effect sizes, and 95% confidence intervals for each comparison for CIELab color parameters of freeze-dried microparticles; Table S5: Summary of ANOVA results including F-statistics, *p*-values, Cohen’s *d* effect sizes, and 95% confidence intervals for each comparison for TPC, TPA and AA in encapsulated materials before and after freeze-drying; Table S6: Summary of ANOVA results and Tukey post hoc Test for biological value and antioxidant potential of microparticles MNPJ during storage; Table S7: Summary of ANOVA results and Tukey post hoc Test for biological value and antioxidant potential of microparticles MNAJ during storage.
