# Supplementary material for: Antioxidant Potential of Jostaberry Phytochemicals Encapsulated in Biopolymer Matrices During Storage
Source: Foods. 2025 Sep 3;14(17):3092. doi: 10.3390/foods14173092 (PMC12428170; doi:10.3390/foods14173092)
Supplement: Supplementary file 1 [file foods-14-03092-s001.zip › Figure S1.pdf]

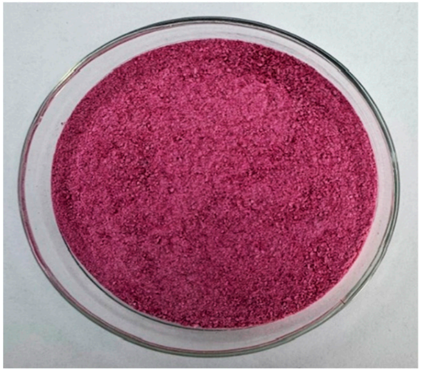

a.

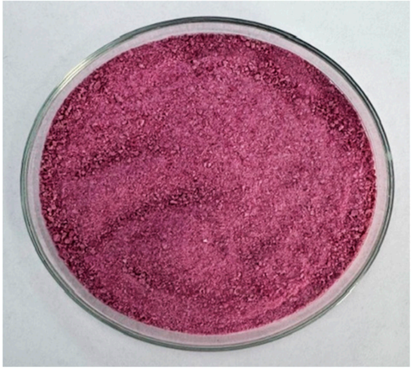

b.

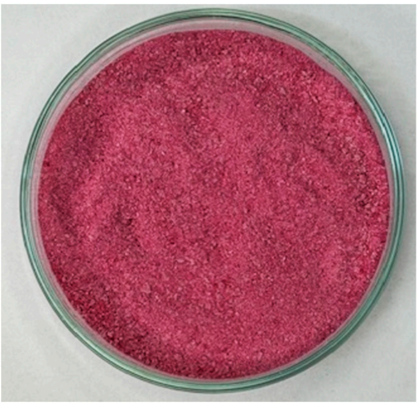

c.

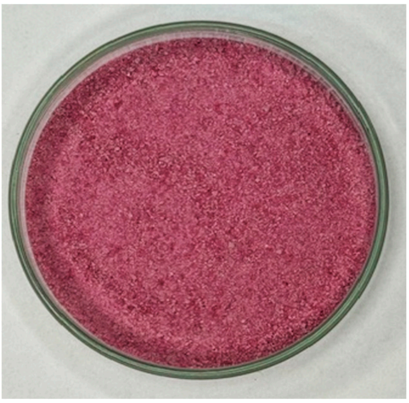

d.

Figure S1. Photos of freeze-dried microparticles

a) MNPJ- josta extract in maltodextrin-nutriose-pectin matrix; b) MNAJ- josta extract in maltodextrin-nutriose-sodium alginate matrix; c),d) $MNPJ_{12}$ and $MNAJ_{12}$- microparticles after 12 months.
