# Supplementary material for: Antioxidant Potential of Jostaberry Phytochemicals Encapsulated in Biopolymer Matrices During Storage
Source: Foods. 2025 Sep 3;14(17):3092. doi: 10.3390/foods14173092 (PMC12428170; doi:10.3390/foods14173092)
Supplement: Supplementary file 1 [file foods-14-03092-s001.zip › Figure S2.pdf]

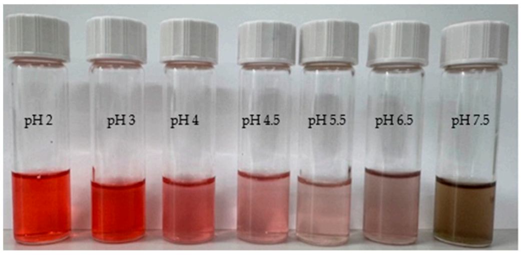

a.

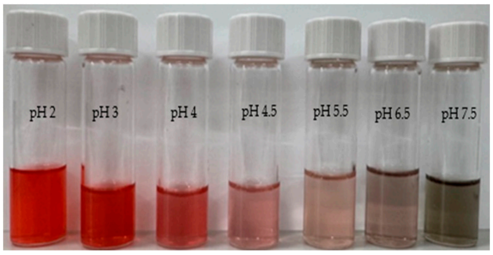

b.

Figure S2. Color change of aqueous solutions of microparticles as a function of pH:
a) MNPJ- josta extract in maltodextrin-nutriose-pectin matrix; b) MNAJ- josta extract in maltodextrin-nutriose-sodium alginate matrix
