# Supplementary material for: Antioxidant Potential of Jostaberry Phytochemicals Encapsulated in Biopolymer Matrices During Storage
Source: Foods. 2025 Sep 3;14(17):3092. doi: 10.3390/foods14173092 (PMC12428170; doi:10.3390/foods14173092)
Supplement: Supplementary file 1 [file foods-14-03092-s001.zip › Figure S3.pdf]

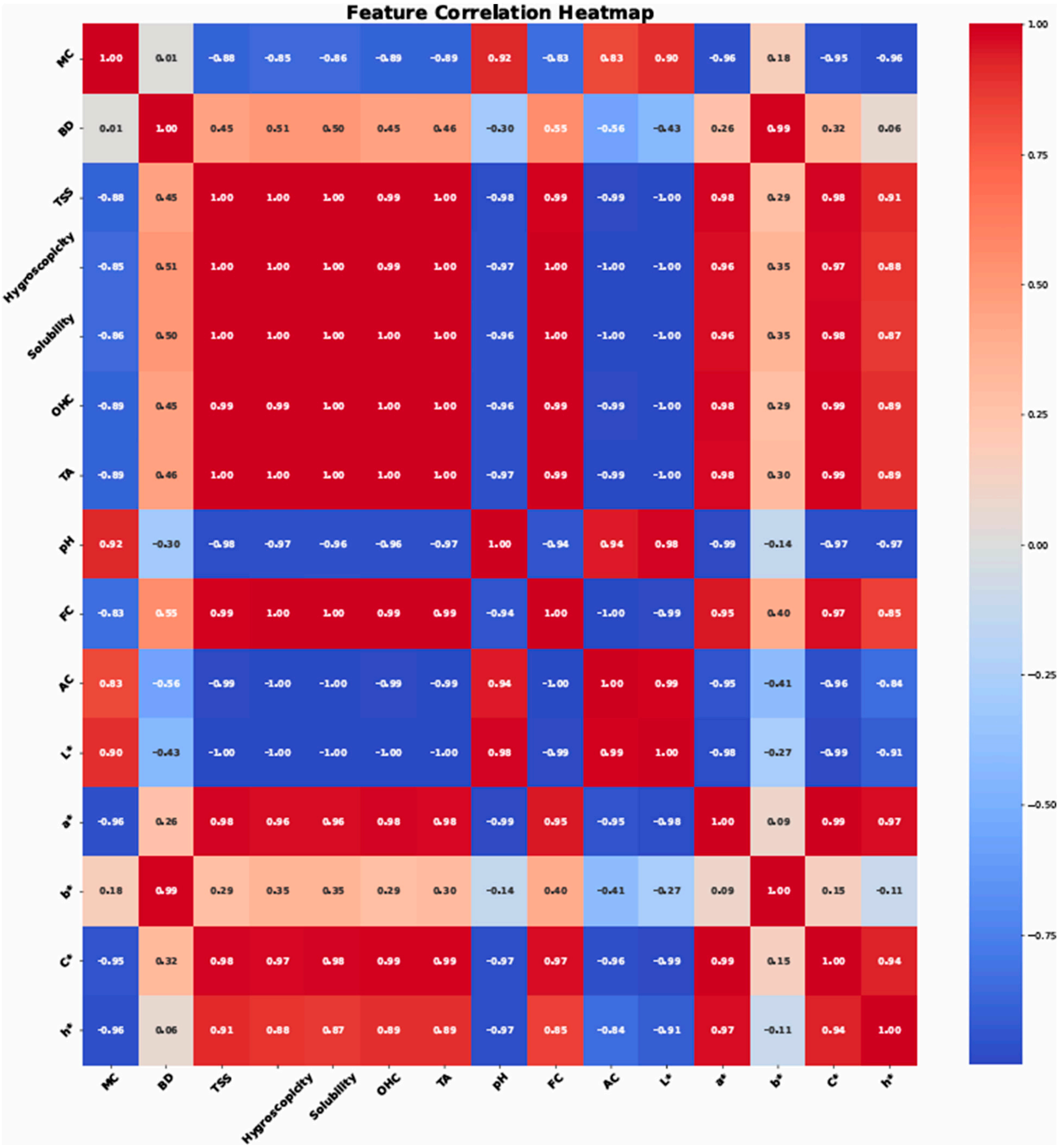

Figure S3. The correlation values between physicochemical characteristics, pH and color parameters in microparticles. FC - fat content; TA - titratable acidity, OHC - oil holding capacity; TSS - total soluble solids; AC – ash content; MC - moisture content; BD - bulk density; L* - lightness; a* - red-green parameter; b* - yellow-blue parameter; C*— chromaticity, h*—hue angle of MNAJ - josta extract in maltodextrin-nutriose-sodium alginate matrix; MNPJ - josta extract in maltodextrin-nutriose-pectin matrix; $MNPJ_{12}$ and $MNAJ_{12}$- microparticles after 12 months of storage.
