# Supplementary material for: Antioxidant Potential of Jostaberry Phytochemicals Encapsulated in Biopolymer Matrices During Storage
Source: Foods. 2025 Sep 3;14(17):3092. doi: 10.3390/foods14173092 (PMC12428170; doi:10.3390/foods14173092)
Supplement: Supplementary file 1 [file foods-14-03092-s001.zip › Table S1.pdf]

Table S1. Summary of ANOVA results including F-statistics, p-values, Cohen's *d* effect sizes, and 95% confidence intervals for each comparison for physicochemical indicators, phytochemical content and AA of hydroethanolic extracts (70%, 1:100 ratio (*m*/*v*)) from frozen and freeze-dried jostaberry

Indices	F statistic	p-value	Cohen's *d*	95% Confidence interval
DW	4,088,151.68	3.59×10^{-13}	−1650.89	(−73.93, −73.73)
FC	11,928.60	4.21×10^{-8}	−89.18	(−1.45, −1.37)
AC	17,579.08	1.94×10^{-8}	−108.26	(−2.82, −2.70)
TPC	170.86	1.98×10^{-4}	−10.67	(−3.27, −2.13)
TFC	445.54	2.98×10^{-5}	17.23	(0.95, 1.23)
TFC	750.00	1.06×10^{-5}	22.36	(0.45, 0.55)
TAC	7,288.22	1.13×10^{-7}	69.71	(4.10, 4.38)
AA by DPPH	19,037.39	1.65×10^{-8}	112.66	(21.23, 22.11)
AA by ABTS	14,919.20	2.69×10^{-8}	99.73	(45.15, 47.25)

FJ—frozen jostaberry; FDJ—freeze-dried jostaberry; DW - dry weight; FC – fat content; AC – ash content; TPC - total polyphenol content; TFC - total flavonoid content; AA - antioxidant activity.
