# Supplementary material for: Antioxidant Potential of Jostaberry Phytochemicals Encapsulated in Biopolymer Matrices During Storage
Source: Foods. 2025 Sep 3;14(17):3092. doi: 10.3390/foods14173092 (PMC12428170; doi:10.3390/foods14173092)
Supplement: Supplementary file 1 [file foods-14-03092-s001.zip › Table S2.pdf]

Table S2. Summary of ANOVA results including F-statistics, p-values, Cohen's *d* effect sizes, and 95% confidence intervals for each comparison for biological active compounds identified and quantified by the HPLC and capillary electrophoresis method in jostaberry hydroethanolic 70% extract (1:100, *m*/*v*) from frozen and freeze-dried jostaberry

Biological active compounds	F statistic	p-value	Cohen's *d*	95% Confidence interval
Anthocyanins	149544.231	2.68×10^{-10}	315.747	(7.992, 8.108)
Ascorbic acid	15552.600	2.48×10^{-8}	101.825	(1.574, 1.646)
Chlorogenic acid	173.400	0.000192	10.752	(0.134, 0.206)
Caffeic acid	24.000	0.00805	4.000	(0.017, 0.063)
Rutoside	630.375	1.49×10^{-5}	20.500	(0.365, 0.455)
Malic acid	1067.077	5.24×10^{-6}	-26.672	(-0.738, -0.622)
Citric acid	3197.400	5.86×10^{-7}	-46.169	(-0.766, -0.694)
Fumaric acid	7561.500	1.05×10^{-7}	-71.000	(-2.198, -2.062)
