# Supplementary material for: Antioxidant Potential of Jostaberry Phytochemicals Encapsulated in Biopolymer Matrices During Storage
Source: Foods. 2025 Sep 3;14(17):3092. doi: 10.3390/foods14173092 (PMC12428170; doi:10.3390/foods14173092)
Supplement: Supplementary file 1 [file foods-14-03092-s001.zip › Table S3.pdf]

**Table S3.** Summary of ANOVA results including F-statistics, p-values, Cohen's *d* effect sizes, and 95% confidence intervals for each comparison for physicochemical indicators of freeze-dried microparticles

| Physicochemical indicators | F statistic | p-value | Cohen's *d* | 95% Confidence interval |
|---|---|---|---|---|
| | | MNPJ and $MNPJ_{12}$ | | |
| MC | 38.473 | 0.003 | -5.064 | (-0.222, -0.085) |
| BD | 2281.500 | $1.15\times10^{-6}$ | -39.000 | (-0.041, -0.037) |
| TSS | 17.043 | 0.015 | 3.371 | (0.031, 0.156) |
| Hygroscopicity | 1.000 | 0.374 | 0.816 | (-0.021, 0.044) |
| Solubility | 170.307 | 0.00020 | 10.655 | (1.482, 2.282) |
| OHC | 112.067 | 0.00045 | 8.644 | (0.302, 0.518) |
| TA | 25.057 | 0.007 | 4.087 | (0.254, 0.886) |
| pH | 96.000 | 0.00061 | -8.000 | (-0.103, -0.057) |
| FC | 0.000 | 1.000 | 0.000 | (-0.023, 0.023) |
| AC | 0.626 | 0.473 | 0.646 | (-0.051, 0.092) |
| | | MNAJ and $MNAJ_{12}$ | | |
| MC | 65.636 | 0.00126 | -6.615 | (-0.170, -0.083) |
| BD | 580.409 | $1.76\times10^{-5}$ | -19.671 | (-0.042, -0.033) |
| TSS | 97.587 | 0.00059 | 8.066 | (0.161, 0.286) |
| Hygroscopicity | 40.692 | 0.003 | 5.208 | (0.022, 0.055) |
| Solubility | 1.781 | 0.253 | 1.090 | (-9.669, 27.566) |
| OHC | 90.353 | 0.00068 | 7.761 | (0.227, 0.413) |
| TA | 52.686 | 0.00191 | 5.927 | (0.352, 0.788) |
| pH | 1536.000 | $2.53\times10^{-6}$ | -32.000 | (-0.343, -0.297) |
| FC | 0.000 | 1.000 | 0.000 | (-0.023, 0.023) |
| AC | 3.692 | 0.127 | 1.569 | (-0.018, 0.098) |

MNPJ - josta extract in maltodextrin-nutriose-pectin matrix; MNAJ - josta extract in maltodextrin-nutriose-sodium alginate matrix. $MNPJ_{12}$ and $MNAJ_{12}$- microparticles after 12 months of storage. MC - moisture content; BD - bulk density; TSS - total soluble solids; OHC - oil holding capacity; TA - titratable acidity; FC - fat content; AC- ash content.
