# Supplementary material for: Antioxidant Potential of Jostaberry Phytochemicals Encapsulated in Biopolymer Matrices During Storage
Source: Foods. 2025 Sep 3;14(17):3092. doi: 10.3390/foods14173092 (PMC12428170; doi:10.3390/foods14173092)
Supplement: Supplementary file 1 [file foods-14-03092-s001.zip › Table S4.pdf]

Table S4. Summary of ANOVA results including F-statistics, p-values, Cohen's *d* effect sizes, and 95% confidence intervals for each comparison for CIELab color parameters of freeze-dried microparticles

Physicochemical indicators	F statistic	p-value	Cohen's *d*	95% Confidence interval
		MNPJ and $MNPJ_{12}$		
L*	17.464	0.0139	-3.412	(-2.447, -0.493)
a*	235.409	0.000105	12.528	(1.720, 2.480)
b*	700.412	1.21×10^{-5}	-21.609	(-1.392, -1.128)
C*	122.880	0.000377	9.051	(0.240, 0.400)
h*	74.483	0.000991	7.047	(0.407, 0.793)
		MNAJ and $MNAJ_{12}$		
L*	128.797	0.000344	-9.266	(-2.626, -1.594)
a*	894.348	7.45×10^{-6}	24.418	(2.477, 2.983)
b*	1266.892	3.72×10^{-6}	-29.062	(-1.348, -1.152)
C*	49.463	0.00215	5.742	(0.157, 0.363)
h*	1499.676	2.66×10^{-6}	31.619	(1.262, 1.458)
