# Supplementary material for: Antioxidant Potential of Jostaberry Phytochemicals Encapsulated in Biopolymer Matrices During Storage
Source: Foods. 2025 Sep 3;14(17):3092. doi: 10.3390/foods14173092 (PMC12428170; doi:10.3390/foods14173092)
Supplement: Supplementary file 1 [file foods-14-03092-s001.zip › Table S5.pdf]

Table S5. Summary of ANOVA results including F-statistics, p-values, Cohen's *d* effect sizes, and 95% confidence intervals for each comparison for TPC, TPA and AA in encapsulated materials until and after freeze-drying

Physicochemical indicators	F statistic	p-value	Cohen's *d*	95% Confidence interval
MNPJ (until freeze-drying/ after freeze-drying)				
TPC	1837.500	1.77×10^{-6}	35.000	(0.327, 0.373)
TAC	258.769	8.73×10^{-5}	13.134	(0.160, 0.227)
AA by DPPH	1190.700	4.21×10^{-6}	28.174	(0.579, 0.681)
AA by ABTS	52785.877	2.15×10^{-9}	187.592	(6.464, 6.622)
MNAJ (until freeze-drying/ after freeze-drying)				
TPC	576.923	1.78×10^{-5}	19.612	(0.442, 0.558)
TAC	768.000	1.01×10^{-5}	22.627	(0.144, 0.176)
AA by DPPH	693.375	1.24×10^{-5}	21.500	(0.385, 0.475)
AA by ABTS	9397.271	6.79×10^{-8}	79.151	(5.012, 5.308)

MNPJ - microparticles of josta extract in maltodextrin-nutriose-pectin matrix; MNAJ – josta extract in maltodextrin-nutriose-sodium alginate matrix; TPC - total polyphenol content; TAC- total anthocyanin content; AA – antioxidant activity.
