# Supplementary material for: Antioxidant Potential of Jostaberry Phytochemicals Encapsulated in Biopolymer Matrices During Storage
Source: Foods. 2025 Sep 3;14(17):3092. doi: 10.3390/foods14173092 (PMC12428170; doi:10.3390/foods14173092)
Supplement: Supplementary file 1 [file foods-14-03092-s001.zip › Table S6.pdf]

Table S6. Summary of ANOVA results and Tukey Post-Hoc Test for biological value and antioxidant potential of microparticles MNPJ during storage

Physicochemical indicators/ ANOVA results	Group 1	Group 2	Mean Diff	p-adj	Tukey CI	Cohen's d	Diff 95% CI
TPC	12 months	3 months	0.54	3.70×10^{-2}	(0.03, 1.04)	-2.57	(-1.01, -0.06)
ANOVA F - 4.10	12 months	6 months	0.31	2.67×10^{-1}	(-0.19, 0.82)	-1.64	(-0.75, 0.12)
ANOVA p - 4.90×10^{-2}	12 months	After production	0.38	1.57×10^{-1}	(-0.13, 0.88)	-2.28	(-0.75, -0.00)
	3 months	6 months	-0.22	5.19×10^{-1}	(-0.73, 0.28)	1.03	(-0.27, 0.72)
	3 months	After production	-0.16	7.38×10^{-1}	(-0.66, 0.34)	0.84	(-0.28, 0.60)
	6 months	After production	0.06	9.77×10^{-1}	(-0.44, 0.57)	-0.36	(-0.46, 0.33)
TPC RE	12 months	3 months	10.75	1.00×10^{-4}	(8.84, 12.67)	-12.49	(-12.70, -8.80)
ANOVA F – 113.65	12 months	6 months	6.27	1.00×10^{-4}	(4.35, 8.18)	-11.16	(-7.54, -4.99)
ANOVA p - 6.74×10^{-7}	12 months	After production	7.57	1.00×10^{-4}	(5.65, 9.48)	-14.51	(-8.75, -6.39)
	3 months	6 months	-4.49	3.00×10^{-4}	(-6.40, -2.57)	5.01	(2.46, 6.52)
	3 months	After production	-3.18	3.20×10^{-3}	(-5.10, -1.27)	3.65	(1.21, 5.16)
	6 months	After production	1.30	2.09×10^{-1}	(-0.61, 3.22)	-2.26	(-2.61, 0.00)
TAC	12 months	3 months	0.23	1.90×10^{-3}	(0.10, 0.36)	-3.53	(-0.38, -0.08)
ANOVA F – 4.10	12 months	6 months	0.23	1.80×10^{-3}	(0.10, 0.36)	-5.02	(-0.33, -0.13)
ANOVA p - 4.90×10^{-2}	12 months	After production	0.30	3.00×10^{-4}	(0.17, 0.43)	-6.24	(-0.41, -0.19)
	3 months	6 months	0.00	1	(-0.13, 0.13)	-0.01	(-0.11, 0.11)
	3 months	After production	0.07	3.57×10^{-1}	(-0.06, 0.20)	-1.36	(-0.19, 0.05)
	6 months	After production	0.07	3.64×10^{-1}	(-0.06, 0.20)	-3.06	(-0.12, -0.02)
TAC RE	12 months	3 months	8.92	1.00×10^{-4}	(6.76, 11.08)	-9.28	(-11.10, -6.74)
ANOVA F – 109.62	12 months	6 months	7.38	1.00×10^{-4}	(5.22, 9.54)	-9.04	(-9.23, -5.53)
ANOVA p - 7.75×10^{-7}	12 months	After production	11.68	1.00×10^{-4}	(9.52, 13.84)	-14.35	(-13.53, -9.84)
	3 months	6 months	-1.54	1.82×10^{-1}	(-3.70, 0.62)	1.84	(-0.36, 3.44)
	3 months	After production	2.76	1.46×10^{-2}	(0.61, 4.92)	-3.31	(-4.66, -0.87)
	6 months	After production	4.30	1.00×10^{-3}	(2.14, 6.46)	-6.48	(-5.81, -2.80)
AA by DPPH	12 months	3 months	0.48	2.70×10^{-3}	(0.20, 0.76)	-47.93	(-0.50, -0.46)
ANOVA F - 17.52	12 months	6 months	0.36	1.47×10^{-2}	(0.08, 0.64)	-22.74	(-0.40, -0.32)
ANOVA p - 7.08×10^{-4}	12 months	After production	0.60	6.00×10^{-4}	(0.32, 0.88)	-3.98	(-0.95, -0.26)
	3 months	6 months	-0.12	5.52×10^{-1}	(-0.40, 0.16)	7.59	(0.08, 0.16)
	3 months	After production	0.12	5.31×10^{-1}	(-0.16, 0.40)	-0.81	(-0.47, 0.22)
	6 months	After production	0.24	9.22×10^{-2}	(-0.04, 0.52)	-1.60	(-0.59, 0.10)

AA by ABTS	12 months	3 months	0.48	1.00×10^{-4}	(0.35, 0.61)	-9.41	(-0.60, -0.36)
ANOVA F – 51.09	12 months	6 months	0.39	1.00×10^{-4}	(0.26, 0.52)	-8.61	(-0.49, -0.29)
ANOVA p - 1.46×10^{-5}	12 months	After production	0.30	4.00×10^{-4}	(0.17, 0.43)	-6.63	(-0.40, -0.20)
	3 months	6 months	-0.09	2.08×10^{-1}	(-0.22, 0.04)	1.63	(-0.04, 0.22)
	3 months	After production	-0.18	1.03×10^{-2}	(-0.31, -0.05)	3.26	(0.05, 0.31)
	6 months	After production	-0.09	2.08×10^{-1}	(-0.22, 0.04)	1.80	(-0.02, 0.20)

MNPJ - josta extract in maltodextrin-nutriose-pectin matrix; MNAJ - josta extract in maltodextrin-nutriose-sodium alginate matrix. TPC - total polyphenol content; RE- retention efficiency; TAC- total anthocyanin content; AA – antioxidant activity.
