# Supplementary material for: Antioxidant Potential of Jostaberry Phytochemicals Encapsulated in Biopolymer Matrices During Storage
Source: Foods. 2025 Sep 3;14(17):3092. doi: 10.3390/foods14173092 (PMC12428170; doi:10.3390/foods14173092)
Supplement: Supplementary file 1 [file foods-14-03092-s001.zip › Table S7.pdf]

Table S7. Summary of ANOVA results and Tukey Post-Hoc Test for biological value and antioxidant potential of microparticles MNAJ during storage

Physicochemical indicators/ ANOVA results	Group 1	Group 2	Mean Diff	p-adj	Tukey CI	Cohen's d	Diff 95% CI
TPC	12 months	3 months	0.14	2.02×10^{-2}	(0.02, 0.26)	-4.81	(-0.21, -0.08)
ANOVA F - 14.21	12 months	6 months	0.10	8.95×10^{-2}	(-0.02, 0.22)	-1.89	(-0.23, 0.02)
ANOVA p - 1.43×10^{-3}	12 months	After production	-0.08	2.61×10^{-1}	(-0.20, 0.04)	1.77	(-0.02, 0.17)
	3 months	6 months	-0.04	7.18×10^{-1}	(-0.16, 0.08)	0.82	(-0.07, 0.15)
	3 months	After production	-0.22	1.70×10^{-3}	(-0.34, -0.10)	6.47	(0.14, 0.30)
	6 months	After production	-0.18	5.90×10^{-3}	(-0.30, -0.06)	3.13	(0.05, 0.31)
TPC RE	12 months	3 months	3.11	5.93×10^{-2}	(-0.12, 6.35)	-2.55	(-5.88, -0.35)
ANOVA F – 6.35	12 months	6 months	2.21	2.05×10^{-1}	(-1.02, 5.45)	-3.30	(-3.73, -0.69)
ANOVA p - 1.64×10^{-2}	12 months	After production	-0.70	8.98×10^{-1}	(-3.93, 2.54)	0.50	(-2.44, 3.83)
	3 months	6 months	-0.90	8.11×10^{-1}	(-4.13, 2.34)	0.84	(-1.53, 3.32)
	3 months	After production	-3.81	2.27×10^{-2}	(-7.04, -0.57)	2.36	(0.15, 7.47)
	6 months	After production	-2.91	7.86×10^{-2}	(-6.14, 0.32)	2.32	(0.07, 5.75)
TAC	12 months	3 months	0.05	3.91×10^{-1}	(-0.05, 0.15)	-1.04	(-0.16, 0.06)
ANOVA F – 13.96	12 months	6 months	0.19	1.10×10^{-3}	(0.09, 0.29)	-6.02	(-0.26, -0.12)
ANOVA p - 1.52×10^{-3}	12 months	After production	0.06	2.50×10^{-1}	(-0.04, 0.16)	-1.69	(-0.15, 0.02)
	3 months	6 months	0.14	8.30×10^{-3}	(0.04, 0.24)	-3.65	(-0.22, -0.05)
	3 months	After production	0.01	9.83×10^{-1}	(-0.09, 0.11)	-0.26	(-0.11, 0.09)
	6 months	After production	-0.13	1.32×10^{-2}	(-0.23, -0.03)	6.63	(0.08, 0.17)
TAC RE	12 months	3 months	3.08	1.70×10^{-2}	(0.61, 5.55)	-4.96	(-4.49, -1.67)
ANOVA F – 14.66	12 months	6 months	-0.50	9.13×10^{-1}	(-2.98, 1.97)	0.42	(-2.21, 3.21)
ANOVA p - 1.29×10^{-3}	12 months	After production	3.59	7.20×10^{-3}	(1.12, 6.06)	-4.70	(-5.32, -1.86)
	3 months	6 months	-3.58	7.30×10^{-3}	(-6.06, -1.11)	3.26	(1.09, 6.07)
	3 months	After production	0.51	9.08×10^{-1}	(-1.96, 2.99)	-0.85	(-1.87, 0.85)
	6 months	After production	4.09	3.20×10^{-3}	(1.62, 6.57)	-3.45	(-6.78, -1.41)
AA by DPPH	12 months	3 months	0.46	1.00×10^{-4}	(0.34, 0.58)	-29.09	(-0.50, -0.42)
ANOVA F – 65.15	12 months	6 months	0.43	1.00×10^{-4}	(0.31, 0.55)	-27.20	(-0.47, -0.39)
ANOVA p - 5.79×10^{-6}	12 months	After production	0.30	2.00×10^{-4}	(0.18, 0.41)	-4.90	(-0.43, -0.16)
	3 months	6 months	-0.03	8.46×10^{-1}	(-0.15, 0.09)	1.50	(-0.02, 0.08)
	3 months	After production	-0.16	9.40×10^{-3}	(-0.28, -0.05)	2.64	(0.02, 0.30)
	6 months	After production	-0.13	2.79×10^{-2}	(-0.25, -0.02)	2.16	(-0.01, 0.27)

AA by ABTS	12 months	3 months	0.55	1.00×10^{-4}	(0.39, 0.71)	-7.78	(-0.71, -0.39)
ANOVA F – 48.86	12 months	6 months	0.38	3.00×10^{-4}	(0.22, 0.54)	-7.45	(-0.50, -0.26)
ANOVA p - 1.73×10^{-5}	12 months	After production	0.50	1.00×10^{-4}	(0.34, 0.66)	-8.33	(-0.64, -0.36)
	3 months	6 months	-0.17	3.91×10^{-2}	(-0.33, -0.01)	2.69	(0.03, 0.31)
	3 months	After production	-0.05	7.57×10^{-1}	(-0.21, 0.11)	0.71	(-0.11, 0.21)
	6 months	After production	0.12	1.58×10^{-1}	(-0.04, 0.28)	-2.35	(-0.24, -0.00)

MNPJ - josta extract in maltodextrin-nutriose-pectin matrix; MNAJ - josta extract in maltodextrin-nutriose-sodium alginate matrix. TPC - total polyphenol content; RE- retention efficiency; TAC- total anthocyanin content; AA – antioxidant activity.
